# *In silico *characterization of the family of PARP-like poly(ADP-ribosyl)transferases (pARTs)

**DOI:** 10.1186/1471-2164-6-139

**Published:** 2005-10-04

**Authors:** Helge Otto, Pedro A Reche, Fernando Bazan, Katharina Dittmar, Friedrich Haag, Friedrich Koch-Nolte

**Affiliations:** 1 Institute of Immunology, University Hospital Hamburg-Eppendorf, Martinistr. 52, 20246 Hamburg, Germany.; 2 DNAX Research Institute, Palo Alto, CA 94304, USA.; 3 Dana-Farber Cancer Institute, Harvard University, Boston, MA 02115, USA.; 4 Depts. of Molecular Biology and Protein Engineering, Genentech, SF, CA 94080, USA.; 5 Department of Integrative Biology, Brigham Young University, Provo, UT 84602, USA.

## Abstract

**Background:**

ADP-ribosylation is an enzyme-catalyzed posttranslational protein modification in which mono(ADP-ribosyl)transferases (mARTs) and poly(ADP-ribosyl)transferases (pARTs) transfer the ADP-ribose moiety from NAD onto specific amino acid side chains and/or ADP-ribose units on target proteins.

**Results:**

Using a combination of database search tools we identified the genes encoding recognizable pART domains in the public genome databases. In humans, the pART family encompasses 17 members. For 16 of these genes, an orthologue exists also in the mouse, rat, and pufferfish. Based on the degree of amino acid sequence similarity in the catalytic domain, conserved intron positions, and fused protein domains, pARTs can be divided into five major subgroups. All six members of groups 1 and 2 contain the H-Y-E trias of amino acid residues found also in the active sites of Diphtheria toxin and Pseudomonas exotoxin A, while the eleven members of groups 3 – 5 carry variations of this motif. The pART catalytic domain is found associated in Lego-like fashion with a variety of domains, including nucleic acid-binding, protein-protein interaction, and ubiquitylation domains. Some of these domain associations appear to be very ancient since they are observed also in insects, fungi, amoebae, and plants. The recently completed genome of the pufferfish *T. nigroviridis *contains recognizable orthologues for all pARTs except for pART7. The nearly completed albeit still fragmentary chicken genome contains recognizable orthologues for twelve pARTs. Simpler eucaryotes generally contain fewer pARTs: two in the fly *D. melanogaster*, three each in the mosquito *A. gambiae*, the nematode *C. elegans*, and the ascomycete microfungus *G. zeae*, six in the amoeba *E. histolytica*, nine in the slime mold *D. discoideum*, and ten in the cress plant *A. thaliana*. GenBank contains two pART homologues from the large double stranded DNA viruses Chilo iridescent virus and Bacteriophage Aeh1 and only a single entry (from *V. cholerae*) showing recognizable homology to the pART-like catalytic domains of Diphtheria toxin and Pseudomonas exotoxin A.

**Conclusion:**

The pART family, which encompasses 17 members in the human and 16 members in the mouse, can be divided into five subgroups on the basis of sequence similarity, phylogeny, conserved intron positions, and patterns of genetically fused protein domains.

## Background

ADP-ribosylation is a posttranslational protein modification in which the ADP-ribose moiety is transferred from NAD onto specific amino acid side chains of target proteins [1-4]. ADP-ribosylation was originally discovered as the pathogenic principle of Diphtheria toxin, a multidomain secreted protein which inactivates elongation factor 2 by ADP-ribosylation after translocation into eucaryotic cells [5]. Subsequently, numerous other bacterial toxins were shown to ADP-ribosylate target proteins in host cells. Moreover, endogenous toxin-like ADP-ribosylating enzyme activities were detected in eucaryotic cells. Several of these enzymes were purified to homogeneity, sequenced, expressed as recombinant proteins, and crystallized.

Sequence and structural analyses revealed the existence of two distinct families of toxin-related ADP-ribosyltransferases in mammals [6,7]: The RT6 family of GPI-anchored and secretory mono-(ADP-ribosyl)transferases (mARTs) catalyzes mono-ADP-ribosylation of cell surface and secretory proteins [8]. The PARP family of nuclear and cytoplasmic poly(ADP-ribosyl)transferases (pARTs) catalyzes poly-ADP-ribosylation of nuclear and cytosolic proteins [9-12]. While mARTs have been implicated to mediate signalling functions of extracellular NAD, pARTs have been shown to play important roles in DNA repair and maintenance of genome integrity [8,9,12].

In this paper we use the term pART (poly ADP-ribosyltransferase) rather than the more established term PARP (poly-ADP-ribosyl-polymerase) for various reasons. Firstly, to emphasize the structural and functional similarities of the poly- and mono-ADP-rib syltransferase subfamilies. Secondly, with respect to the biochemical classficiation of enzymes the term transferase is more appropriate than polymerase: ADP-riboslytransferases belong to the family of glycosyltransferases; the term polymerase is more commonly used for template-dependent DNA or RNA synthesizing enyzmes. Thirdly, use of the term PARP would have confounded comparison of our results with those of the recent review by Ame *et al*. [11], who used the term PARP and a numbering system without regard to structural similarities among gene family members.

**Figure 1:**
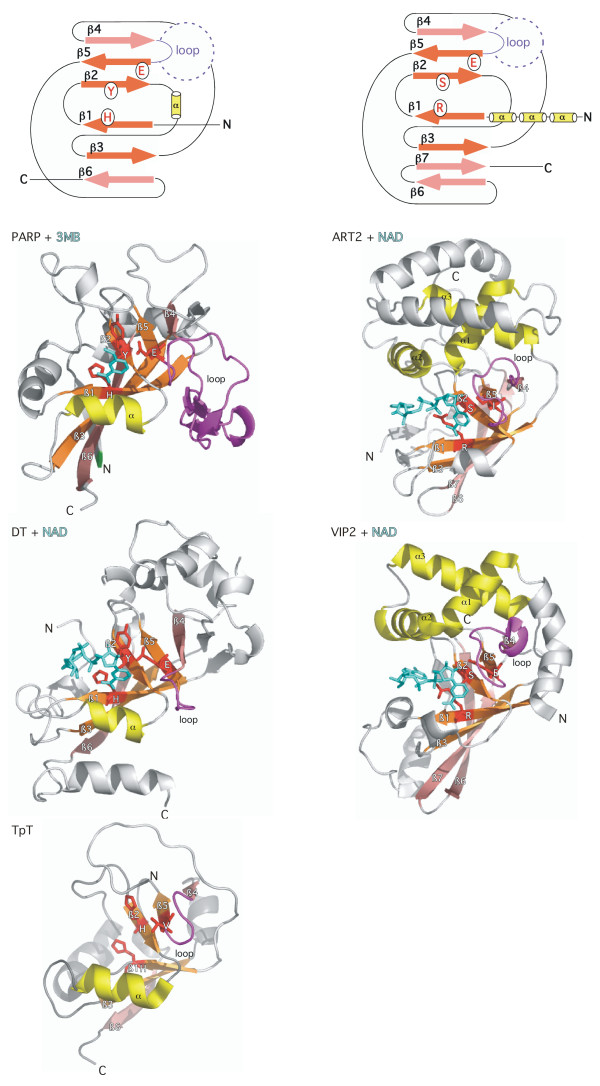
**Schematic illustration of the distinguishing structural features of the PARP-1/DT vs. the ART2/VIP2 subfamilies of ADP-ribosyltransferases. **Two abutting sheets of anti-parallel β strands form the upper and lower jaws of a Pacman-like NAD-binding crevice in all known structures of ADP-ribosyltransferases. The distinguishing structural features of the PARP/DT and ART2/VIP2 subfamilies are depicted schematically on top and are highlighted in the structures of chicken PARP-1 (3pax), diphtheria toxin (DT) (1tox), an archael tRNA:NAD 2'-phosphotransferase (TpT) (1wfx), rat ART2 (1og3) and *B. cereus *VIP2 toxin (1qs2) below. The structures are depicted from the "front view" with a full view of the ligands bound in the active site crevice. The ligands NAD and 3MB are colored cyan and are depicted as stick models. The central four β-strands (from top to bottom: β 5, β 2, β 1, β 3, colored orange) are conserved in all mARTs and pARTs. The β strands at the edges of the respective sheets (β 4 and β 6, colored pink) show greater structural variation than the central β strands. The H-Y-E motif residues are depicted in red and their side chains are shown as sticks. The glutamic acid residue at the front edge of β 5 is the critical catalytic residue in both diphtheria toxin and PARP-1 – a corresponding glutamic acid residue is observed also in the 3D structures of rat ART2 and numerous bacterial mARTs. Diphtheria toxin (1tox), pseudomonas exotoxin A (1aer), PARP-1 (3pax), and PARP-2 (1gs0) share the following structural features which are not conserved in either rat ART2 (1og3) or most other bacterial mARTs: the orientation of β 6, the alpha helix between β 2 and β 3 (colored yellow) and the conserved histidine and tyrosine amino acid residues in β 1 and β 3. The loop between β 4 and β 5 (colored magenta) is thought to play a role in the recognition of target proteins and ADP-ribose polymers. Distinguishing features of ART2, VIP2, iota toxin (1gir), and the C3 exoenzymes (1g24, 1ojz) include three conserved alpha helices upstream of β strand 1, a seventh β strand that displaces β strand 6 and an R-S-E- motif instead of the H-Y-E motif of PARP-1 and DT. (Note that the depicted ART2 structure carries a site directed mutation of the catalytic glutamic acid residue E189I). The recently determined 3D structure of the tRNA:NAD 2'-phosphotransferase (1wfx) bears striking resemblance to that of DT and PARP-1 and carries an H-H-V variant of the H-Y-E motif. Note that the structure of the diphtheria toxin catalytic domain shown here in complex with NAD is truncated C-terminally at the proteolytic cleavage site that separates this domain from the translocation domain. The PARP-1 catalytic domain shown here is truncated N-terminally at the position of the phase 0 intron that separates this domain from a neighboring helical domain. The TpT catalytic domain is truncated N-terminally at the point of fusion to a winged-helix domain.

The 3D-structures of rat ART.2 (PDB accession number 1og3), chicken PARP-1 (1a26, 3pax), mouse PARP-2 (1gs0), and numerous ADP-ribosylating toxins uncovered a common NAD binding fold with a conserved core of five β strands arranged in two abutting β sheets [13-19]. These two β sheets form the upper and lower jaws of a Pacman-like active site crevice (Figure 1). Remarkably, only a single amino acid residue, the catalytic glutamic acid residue at the front edge of the fifth conserved β-strand, is strictly conserved in all known 3D structures of enzymatically active mARTs and pARTs. In a seminal study, Collier and co-workers pinpointed the corresponding glutamic acid residue in PARP-1 (before its 3D structure was solved) on the basis of barely detectable sequence similarity to Diphtheria toxin [20,21]. More recently, the 3D structures of anthrax lethal factor, VIP2, and iota toxin have been discovered to harbour ART-domains that lack a corresponding glutamic acid residue and may represent inactivated enzymes [16,22,23].

Comparative structure and amino acid sequence analyses revealed that PARP-1 and PARP-2 share additional secondary structure and conserved amino acids with Diphtheria toxin and Pseudomonas exotoxin A, which evidently are not conserved in other mARTs (Fig. 1) [6,7]. These additional elements include a sixth β strand, an alpha helix between β strands 2 and 3, and a trias of amino acids, the so-called H-Y-E motif, encompassing a histidine resdiue in β strand 1, a tyrosine residue in β strand 3 and the catalytic glutamic acid residue at the front edge of β strand 5. These features, highlighted in the 3D structures of PARP-1 and Diphtheria toxin in Figure 1, clearly distinguish the structures of PARP-1, PARP-2, and DT/ETA from those of a second major ART subfamily that includes rat ART2 and the *Bacillus cereus *VIP2 toxin. Distinguishing features of the ART2/VIP2 subfamliy include a seventh β strand that displaces β strand 6, three conserved alpha helices preceding β strand 1, and an R-S-E trias of amino acid residues in place of the H-Y-E motif of PARP-1 and DT. Interestingly, the recently reported 3D-structure of a prototype member of the family of tRNA:NAD 2' phosphotransferases (TpT) [24] revealed a striking resemblance to the structures of the PARP-1/DT subfamily rather than to those of the ART2/VIP subfamily, including the sixth β strand, the alpha helix between β strands 2 and 3, and a variant H-Y-E motif (H-H-V). These enzymes catalyze removal of a splice junction 2' phosphate from ligated tRNA. This reaction resembles the reaction catalyzed by ARTs but yields ADP-ribose 1"-2" cyclic phosphate rather than ADP-ribosylated proteins [25].

The remarkable degree of plasticity of ART amino acid sequences poses a challenging problem for genome data base mining [7] and even the most sensitive database search programs fail to connect all known members of the ART gene family. Notwithstanding, the results of such *in silico *analyses can provide important insight into the structural and phylogenetic relationship of ART subfamilies. We have previously demonstrated that the known members of the mART gene family in the human and mouse could be faithfully connected with many known bacterial ADP-ribosylating toxins, but not with pARTs or Diphtheria toxin [26,27]. These analyses also pointed out the presence of mART-encoding genes in the genomes of many but not all other model organisms. Of note, no mART-encoding genes could be detected in plants, fungi, or archaea. Here we provide an in depth analysis of the pART gene family.

## Results and discussion

### Identification of human and mouse pART family members in the EST database

The human and mouse pART gene family members were identified using a combination of data base search tools. The human and mouse EST databases as well as the nonredundant GenBank database (nr) were screened with tBLASTn using as queries the amino acid sequences of the catalytic domains of the known and newly identified pART family members. Whenever possible, the full coding sequence of the catalytic domain and of the adjacent regions was assembled using the sequences of published cDNAs and overlapping ESTs. Screening of the EST and nr databases was initiated in 1997 and was repeated in regular intervals. The coding sequences were extended when suitable new sequences became available. When the sequences of the human, mouse and rat genomes were published in 2000, 2001, and 2004, respectively, the EST database searches were complemented with corresponding tBLASTn and BLASTn searches of the genome sequences [28-30]. Thereby, 17 pART family members were identified in the human. These genes were designated pART1-pART17. Numbering reflects the degree of amino acid sequence similarity to PARP-1 (= pART1) and the degree of similarity within each of the pART subgroups. An orthologue for each of these genes was detected in the mouse and in the rat, with the sole exception of pART7.

**Figure 2:**
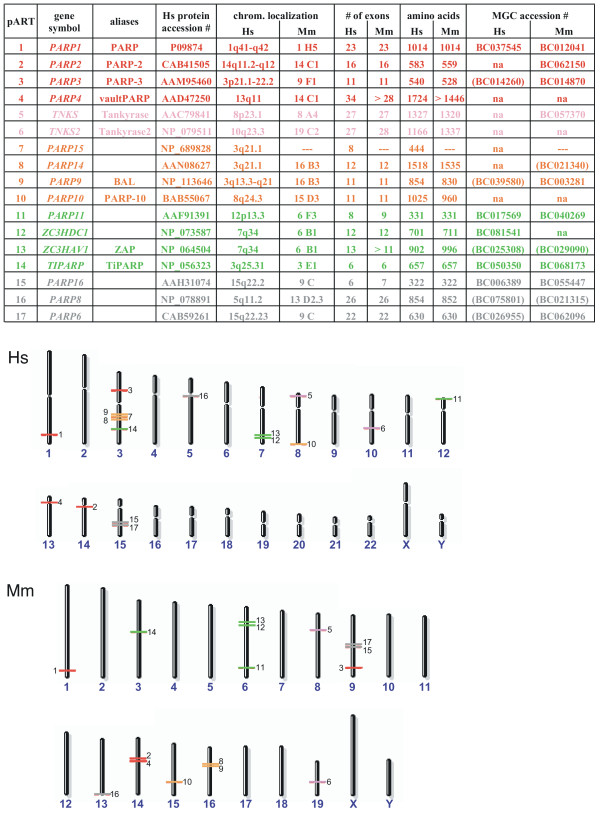
**Chromosomal localizations and exon compositions of the human and mouse pART family members. **A) pART family members are sorted by subgroup on the basis of similarities in amino acid sequence, intron positions and associated protein domains. Color-coding of subgroups is as follows: 1 = red, 2 = pink, 3 = orange, 4 = green, 5 = grey. This color-coding is used in subsequent figures. Official gene designations, common aliases and accession numbers are shown. Exon compositions and lengths of open reading frames are given for the longest known or predicted gene transcripts. Available full length cDNAs from the Mammalian Gene Collection (MGC) are indicated with their respective accession numbers. MGC cDNAs which apparently do not contain the full open reading frame are indicated in parentheses. Hs = *Homo sapiens*, Mm = *Mus musculus*. B) Chromosomal localizations of pART genes were determined by tBLASTn searches of the respective genome sequences using the amino acid sequences of the catalytic domains of individual pARTs. Members of the five pART family subgroups are color-coded as in A).

A complete list of human pART family members, including the common names and aliases of known genes, is presented in Figure 2. Based on the degree of amino acid sequence similarities, conserved intron positions, and fused protein domains, the mammalian pART family can be divided into five major subgroups. Group 1 (pART1-pART4) contains PARP and its closest relatives, PARP-2, PARP-3 and VPARP. Group 2 (pART5, pART6) contains tankyrase 1 and tankyrase 2. Group 3 (pART7-pART10) contains four proteins including the recently described B-Aggressive Lymphoma Protein (BAL = pART9) [31] and a myc-interacting protein with PARP activity (PARP-10) [32]. Group 4 (pART11-pART14) contains four proteins including the recently described Zinc-finger Antiviral Protein (ZAP = pART13) [33] and TCDD-inducible PARP (TiPARP) [34]. Group 5 (pART15-pART17) contains three proteins of unknown function.

The steady growth in the number of matching ESTs obtained for each of the human pART gene family members over the past 6 years is illustrated in additional file 1 ("Representation of pART gene transcripts in the database of expressed sequence tags"). By October 2004, each human pART except pART7 was represented by more than 100 ESTs. Interestingly, each pART except pART7 is represented by more ESTs than poly (ADP-ribose) glycohydrolase (PARG), the single known enzyme capable of removing poly-ADP-ribose from pART target proteins. The large number of ESTs corresponds to a large variety of tissues found to contain pART ESTs and presumably reflects an ubiquitous pattern of gene expression, i.e. akin to that of the house keeping enzymes hypoxanthine-guanine phosphoribosyltransferase (HPRT) and glyceraldehyde-3-phosphate dehydrogenase (GAPD). For comparison, the members of the mART gene family (ART1-ART5), which exhibit highly restricted patterns of expression, are each represented by much fewer ESTs than the pARTs. As of January 2005, the mammalian gene collection  contains annotated full-length cDNA sequences for 10 of the 17 human pARTs and for 12 of 16 mouse pARTs (Fig. 2).

### Chromosomal localizations and exon/intron structures of the human and mouse pART gene family members

The results of tBLASTn and BLASTn searches of the human, mouse, and rat genome sequences yielded the chromosomal localization and the exon/intron structure of each pART gene family member. The chromosomal localizations of the pART genes are represented schematically in Figure 2. All human and mouse pART orthologues lie in regions of conserved synteny. There are three conserved pART gene clusters containing two related paralogues (pARTs 8 and 9; pARTs 12 and 13; pARTs 15 and 17). However, the two most closely related pairs of pARTs (pARTs 5 and 6; pARTs 16 and 17) each are located on different chromosomes. All other pARTs are distributed as single copy genes on different autosomes. In the human genome, the cluster containing pARTs 8 and 9 also contains pART7. Additional file 2 illustrates the local chromosomal environment of this pART gene cluster on human chromosome 3q and the syntenic region on mouse chromosome 16B3. The local order of genes is similar in the human and mouse. However, the region corresponding to pART7 is missing in the mouse. The corresponding region is also missing in the rat genome (not shown).

**Figure 3:**
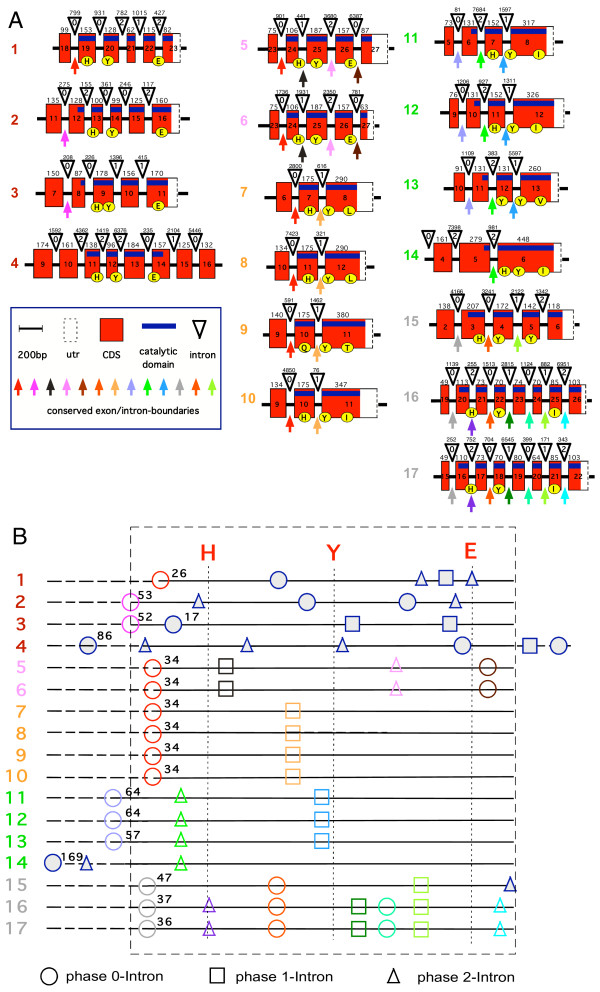
**Schematic diagram of the exon/intron structures of the regions encoding the catalytic domain of pART family members. **A) Exon/intron structures were determined by BLASTn searches of the human genome sequence with individual pART cDNA sequences. Only the exons corresponding to the catalytic domain of PARP-1 are shown. The coding region is marked in red, the 3' untranslated region (utr) is marked in white, and a blue bar marks the region corresponding to the catalytic domain. Exons are represented as boxes with the width of each box reflecting the size of the respective exon (the 3' utr is not drawn to scale). Exon numbers are given with exon 1 corresponding to the exon encoding the presumptive initiation methionine. In all cases except pART4 (VPARP) the catalytic domain is encoded by the 3' terminal exons. Exon sizes (or size of coding region in case of the 3' exons) in basepairs are indicated on top of the boxes. Introns are depicted as triangles and are not drawn to scale. Intron sizes in base pairs are indicated on top of the triangles. The position of each intron with respect to the reading frame is indicated in the triangles (0 = between codons, +1 = between codon positions 1 and 2, +2 = between codon positions 2 and 3). Conserved exon boundaries are marked by colored arrows. Codons corresponding to the H-Y-E motif in the NAD binding crevice of DT and PARP-1 (see Fig. 1) are marked by yellow circles. B) The catalytic domain as delineated in this paper is indicated by the dashed rectangle. For each pART the cDNA coding region within the catalytic domain is marked by a straight line, regions extending beyond this domain in the 5' direction (and in the 3' driection in case of pART4) are marked by dashed lines. The positions of the codons corresponding to the H, Y, E residues in the NAD-binding crevice are indicated by vertical lines. Intron phases are indicated by circles (phase 0), boxes (phase 1), and triangles (phase 2). Numbers indicate the distance in codons between the conserved histidine in β 1 and the next upstream phase 0 intron. Color-coding of conserved introns corresponds to that shown in A). Nonconserved introns are indicated in blue (filled) icons.

The total number of exons in each pART gene is depicted in Figure 2 and the exon structure of the catalytic domain is illustrated schematically for the human pARTs in Figure 3. All intron positions within the coding region are fully conserved in human and mouse orthologues. With the sole exception of pART4 (VPARP), the catalytic domain is encoded by the 3' terminal exons. Remarkably, in all pART genes, with the exception of pART4 (VPARP) and pART14 (TiPARP), the exons encoding the catalytic domain are separated from the rest of the respective coding exons by a phase 0 intron shortly upstream of the codon for the first residue of the conserved H-Y-E catalytic site motif, the conserved histidine in β 1 (Fig. 3). For most pARTs, the amino acid sequences encoded by exons upstream of this phase 0 intron do not show any detectable similarities, except for members of a particular subgroup. We used the position of this phase 0 intron in pART1 to delineate the N-terminal border of the catalytic domain (e.g., see the green labeled end of the PARP-1-model in Figure 1 and the dashed rectangle in Figure 3B).

The exon/intron structures of the pART catalytic domains reveal a number of intriguing features (Fig. 3). The region encoding the catalytic domain is disrupted by a remarkable variety of introns with the number of introns varying from one in subgroup 3 and in pART14 to six in pARTs 16 and 17. The catalytic domain of pART1 (PARP-1) and those of its closest relatives in subgroup 1 are disrupted by three (pARTs 3 and 4) or four (pARTs 1 and 2) introns. Strikingly, not one of these 14 intron positions is conserved. The catalytic domains of the two closely related tankyrases in subgroup 2 each are interrupted by three conserved introns. In subgroup 3, the catalytic domains of pARTs 7–10 each contain a single conserved intron. The pARTs of subgroup 4 (pARTs 11–14) share a single conserved intron in their catalytic domains, pARTs 11–13 share a second conserved intron in the catalytic domain, which is missing in pART14. The pARTs of subgroup 5 (pARTs 15–17) share two conserved introns in their catalytic domains, pARTs 16 and 17 share four additional conserved introns in the catalytic domain, which are missing in pART15.

### Conserved structural features revealed by multiple amino acid sequence alignments and secondary structure predictions

**Figure 4:**
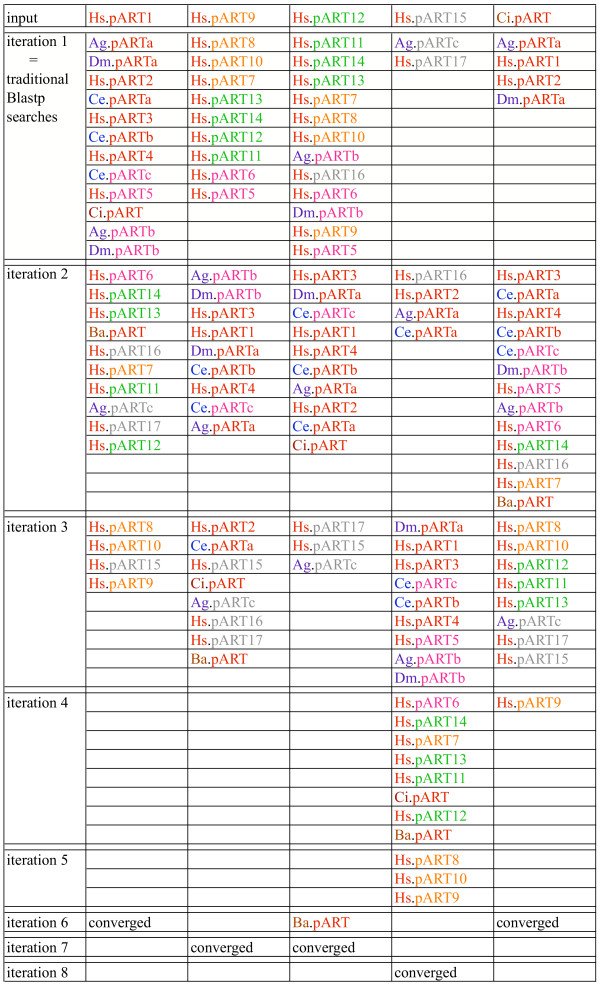
**Representative tiling paths of PSI-BLAST searches initiated with the catalytic domain amino acid sequences of selected pART family members. **PSI-BLAST searches were initiated with the catalytic domain amino acid sequences of the pARTs indicated on top as query sequences with the default threshold setting for the expect value of 0.005. Matching sequences from selected model organisms are indicated at the iteration in which they first appeared above threshold. pART subgroups are color coded as in Figure 2. Accession numbers of the indicated pARTs are listed in Figures 2 and 9. Species of origin is color-coded in the two letter abbreviation of the organism as follows: *Homo sapiens *(Hs) red, *Drosophila melanogaster *(Dm) and *Anopheles gambiae *(Ag) purple, *Caenorrhabditis elegans *(Ce) blue, Chilo iridescent virus (Ci) and Bacteriophage Aeh (Ba) brown.

PSI-BLAST is a powerful, position sensitive iterative program designed to detect distantly related proteins in the protein database [35]. Initial matches in the first iteration correspond to those detected by classic BLASTp searches and typically reveal proteins with an amino acid sequence identity to the query sequence of > 30%. PSI-BLAST then derives a position specific scoring matrix from the aligned protein sequences obtained in the first iteration, which is then used for the subsequent search of the protein database. This process is repeated in an iterative fashion until no further matches are detected and the search 'converges'. We performed PSI-BLAST searches of the protein database using as query the amino acid sequences of the catalytic domain of each member of the pART gene family. Figure 4 schematically illustrates the tiling paths of PSI-BLAST searches obtained with the stringent default threshold setting (0.005 for the expect value) for a representative member of pART family subgroups 1, 3, 4 and 5. Typically, the other members of the same subgroup were detected in the first iteration and obtained the highest scores. The pARTs of other subgroups were usually detected within two additional iterations, except in case of pART15. Here, five iterations were required to detect all pART family members.

The amino acid sequence alignments generated by PSI-BLAST typically contained the highest degree of sequence similarity in secondary structure motifs corresponding to the NAD-binding cores in the known 3D structures of chicken PARP-1 (1a26) and mouse PARP-2 (1gs0). Separate multiple amino acid sequence alignments were generated with T-Coffee for each of the pART subgroups using the orthologous sequences from human and mouse [36]. PSIPRED was used to predict secondary structure units and GenTHREADER was used to predict the optimal alignment of pART amino acid sequences with the 3D structures of chicken PARP-1 and mouse PARP-2 [37]. In all cases, predictions and alignments yielded consistent results with respect to the sole alpha helix and five of the six β-strands of the PARP-1 catalytic domain (see additional files 3, 4, 5, 6, 7: "Multiple amino acid sequence alignments, secondary structure predictions and threading results for pART subgroups 1–5"). The small β strand (β 4) at the upper edge of the active site crevice was aligned and predicted congruently only for subgroups 1–4, and could not be predicted with confidence for the most distant relatives of PARP-1 (pARTs 15–17). Regions corresponding to connecting loops showed significant sequence identities only for members of a particular pART subgroup. Most likely, these regions fold similarly only in closely related pART family members.

A striking result of the alignment analyses is that the H-Y-E catalytic site motif is fully conserved only in subgroups 1 and 2 (pARTs 1–6). All other pARTs show deviations from this motif. The histidine in β 1 is conserved in 9 of the 11 members of subgroup 3–5, the tyrosine in β 3 is conserved in all family members, yet the presumptive catalytic glutamic acid at the N-terminal end of β 6 is exchanged in each of the pARTs 7–17.

Moreover, the amino acid sequence of the loop immediately upstream of β 5 and the active site glutamic acid residue deviates markedly from those of PARP-1 and PARP-2 in most other family members except for the tankyrases (pARTs 5 and 6). A growing body of evidence indicates that this region influences the target specificity of pARTs and mARTs [38-40]. In the 3D structure of PARP-1 with carba-NAD (3pax), the ligand was found to interact with this loop outside of the active site crevice, and it was proposed that this may reflect the binding of the ADP-ribose polymer in the target protein [14].

**Figure 5:**
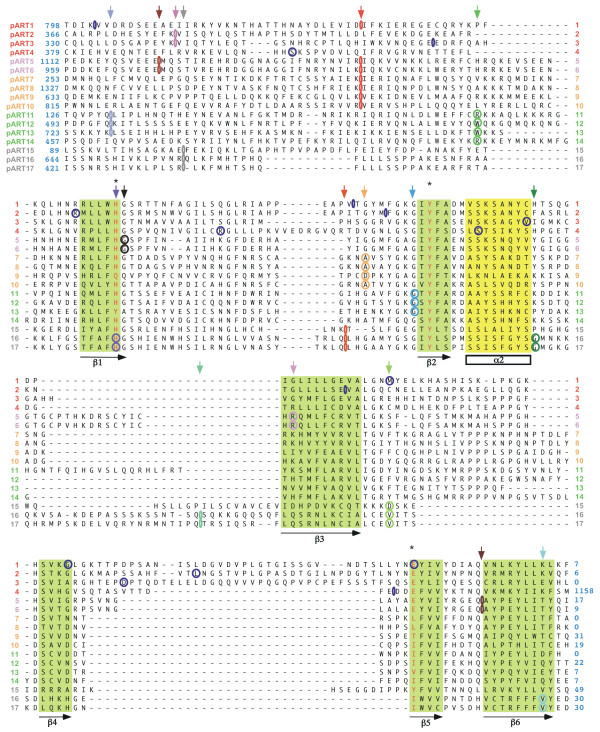
**Multiple amino acid sequence alignment of the catalytic cores of the human pART family. **The multiple sequence alignment was generated with T-Coffee and manually adjusted using the results of the PSI-BLAST, PSIPRED, and GenTHREADER analyses. Numbers at the sequence ends indicate the number of additional residues upstream and downstream of the alignment shown. Residues corresponding to the H Y E motif in the NAD binding crevice of diphtheria toxin are in red and marked by asterisks. The conserved β sheets and alpha helix are shaded in green and yellow. Conserved intron positions are marked in the multiple alignment using the same color-coding as in Figure 3. Conserved intron positions are indicated also above the alignment with arrows. Non-conserved intron positions are marked in blue in the alignment.

The results of the secondary structure prediction and threading analyses were used to refine a multiple amino acid sequence alignment of the catalytic domains of all human pART family members. The resulting alignment is shown in Figure 5. The conserved secondary structure units corresponding to the catalytic NAD binding core (the six beta strands and one alpha helix marked in Figure 1) are indicated schematically below the alignment. The corresponding amino acid residues are highlighted in the alignment. Intron positions are projected onto the amino acid sequence in Figure 5. The positions of conserved introns are marked by colored arrows below the alignment. Note that the alignment diverges most strongly both in length and in sequence in the loops immediately downstream and upstream of β 3.

**Figure 6:**
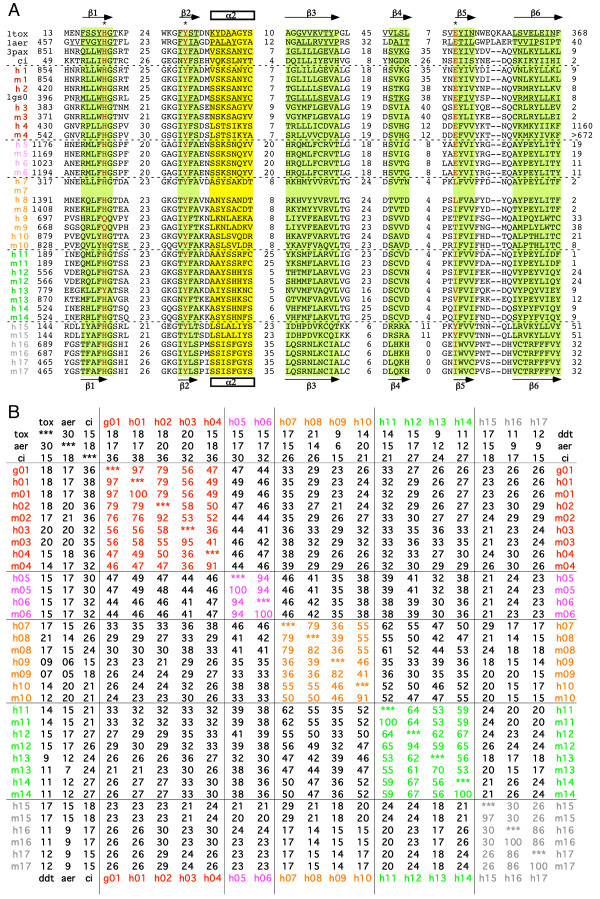
**Structure based amino acid sequence alignment of the catalytic cores of the pART gene family. **A) The alignment is restricted to those regions corresponding to the conserved secondary structure units of PARP-1 and DT as highlighted in Figure 1. The H Y E motif is marked by asterisks and is highlighted in red. Black numbers indicate amino acid residues from the N- and C-terminal ends of the protein and within the loops connecting the structure units shown. For proteins with known 3D structures the pdb accession number is given and the residues corresponding to respective secondary structure units are underlined. 1tox = diphtheria toxin; 1aer = pseudomonas exotoxin A, 3pax = chicken PARP-1 (pART1), 1gs0 = mouse PARP-2 (pART2). Human and mouse pARTs are indicated by colored numbers. The sequence of the putative pART from Chilo iridescent virus is also shown for comparison (ci). B) Pairwise percentage sequence identities were calculated for the 66 amino acid residues shown in A), which correspond to the conserved core secondary structure units in Figure 1.

Figure 6A shows a condensed version of the alignment in which the diverging intervening loops are indicated only by the number of amino acid residues. These 66 amino acid residues can be superimposed well in the 3D structures of PARP-1, PARP-2, DT, and ETA. The respective amino acid sequences of DT, ETA and the putative Chilo iridescent virus pART are also shown for these regions. Figure 6B shows the calculated amino acid sequence identities of the pART family members in this region. The percentage amino acid sequence identity in the aligned core region is higher among members of a particular subgroup than between members of different subgroups, lending support to the subgroup assignments. For each pART, the next most closely related paralogue is a member of the same subgroup. Note that two pairs of pART paralogues show very close sequence similarity: pARTs 5 and 6 (94% identity in the aligned core region) and pARTs 16 and 17 (86% identity). This close similarity is reflected also in the conserved exon intron structures of the respective pART pairs (see Fig. 3).

Comparison of mouse and human pART orthologues shows that seven of such pairs exhibit 100% sequence identity in the aligned core region (pARTs 1, 5, 6, 11, 14, 16, and 17) and six show > 90% identity (pARTs 2, 3, 4, 10, 12, and 15). The mouse and human orthologues of pARTs 8, 9, 13 show the least degrees of sequence identity in this region (82%, 82%, and 70%, respectively) (Fig. 6B).

**Figure 7:**
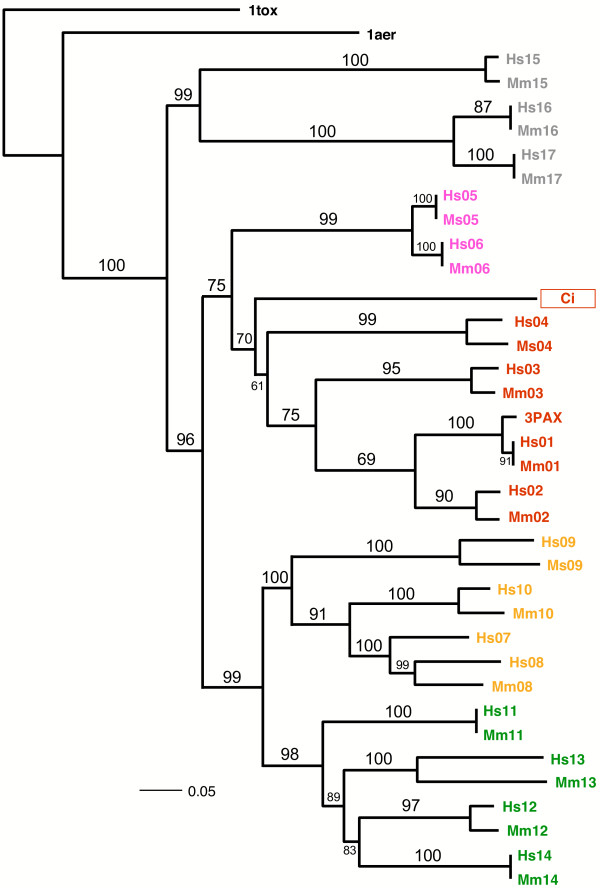
**Phylogram of the evolutionary relationship of the pART family. **Evolutionary relationships of the amino acid sequences in the catalytic core of the pARTs shown in Figure 6 are illustrated as a maximum *a posteriori *phylogram (MAP) of Bayesian Markov Chain Monte Carlo analysis (pP = 0.92). Posterior probabilities were converted into percentages and are shown above the branches. Members of the five pART family subgroups are color-coded as in Figure 2: subgroup 1 = red, 2 = pink, 3 = orange, 4 = green, 5 = grey. Hs = *Homo sapiens*, Mm = *Mus musculus*.

Phylogenetic analysis of the amino acid sequences of the catalytic cores of pARTs resulted in three very similar trees when using Maximum Parsimony (PAUP), Maximum Likelihood (PhyML), and Bayesian Markov Chain Monte Carlo (MrBayes) optimization criteria (Figure 7). All topologies showed moderate to high support values for the recovered relationships. All trees recovered five basic clades corresponding to the subgroups 1–5. The results indicate that pARTs of subgroups 1 and 2 are more closely related (sistergroups) to one another than to members of the other subgroups. A similar relationship is seen for pARTs of subgroups 3 and 4. Note that the putative Chilo iridescent virus pART clusters with the mammalian pARTs of subgroup 1, suggesting that this large double stranded DNA virus may have acquired its pART by horizontal gene transfer.

### The pART catalytic domain has become genetically fused to a wide spectrum of protein domains

With the exception of closely related members within a subgroup, the amino acid sequence similarity between pART family members breaks off upstream of β 1. Interestingly, loss of sequence similarity correlates well with the presence of a phase 0 intron upstream of β 1. All pART family members except pART4 and pART14 contain such a phase 0 intron 26–64 codons upstream of the conserved histidine in β 1 (Fig. 3B).

**Figure 8:**
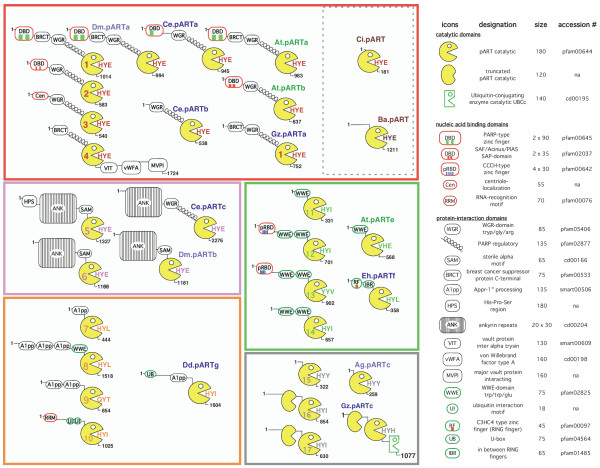
**Schematic diagram of the domain structures of human pARTs and pARTs from distantly related organisms. **Recognizable protein domains in the pART family are represented by the icons defined on the right. The domain structures of human pARTs (on the left, numbered Pacman icons) and related pARTs from other species are illustrated schematically. Potential DNA binding domains are boxed in red, potential ubiquitylation motifs are boxed in green. Members of the five pART family subgroups are grouped within colored boxes using the color-coding as in Figure 2: subgroup 1 = red, 2 = pink, 3 = orange, 4 = green, 5 = grey. Amino acids corresponding to the HYE catalytic site motif of DT and PARP-1 are shown in the mouths of the Pacman icons. Black numbers indicate protein lengths in number of amino acids. Species of origin is color-coded in the two letter abbreviation of the organisms as in Figures 4 and 9: *Drosophila melanogaster *(Dm) and *Anopheles gambiae *(Ag) purple, *Caenorrhabditis elegans *(Ce),* Dictyostelium discoideum *(Dd)*, Entamaoeba histolytica *(Eh), and *Gibberella zeae *(Gz) blue, *Arabidopsis thaliana *(At) green, Chilo iridescent virus (Ci) and Bacteriophage Aeh (Ba) brown. Protein database accession numbers for the illustrated pARTs are listed in Figures 4 and 9. On the right, the approximate size of each domain is indicated in number of amino acid residues. The accession numbers of the respective domain families in the pfam, cd, and smart databases are indicated. In case of zinc finger (zf) containing domains, the number of recognizable zinc fingers is indicated by colored bars within the icon.

Using the sequences flanking the catalytic domain of each pART family member as queries, we performed further PSI-BLAST analyses and searches of the Conserved Domain Database [41]. The results, summarized in Figure 8, reveal that each of the 17 human pARTs with the possible exception of pART15 is a multi-domain protein. Strikingly, the pART catalytic domain is associated – in a Lego like fashion – with a broad spectrum of known protein domains. In all family members except pART4 the catalytic domain represents the C-terminal domain.

A number of associated domains occur in two or more human pART family members. Note that domain sharing generally is restricted to members of a particular pART subgroup. For example, all members of subgroup 1 contain a helical domain preceding the catalytic domain, whereas this domain is missing in members of other pART subgroups. The two members of subgroup 2 share SAM and ankyrin-repeat domains. Three of four pARTs in subgroup 3 share A1pp domains [42], all members of subgroup 4 share WWE domains, and two members of subgroup 5 contain a second, truncated pART domain, reminiscent of the duplicated inactive ART domain found in the VIP2 and iota mART toxins [16,23].

Several pARTs carry recognizable zinc-fingers containing putative RNA-, DNA-, or ubiquitin-binding domains (pART1, pART2, pART10, pART12, pART13). This indicates that the genetic fusion of a pART catalytic domain with zinc-fingers has occurred repeatedly in evolution.

### Representation of pARTs in other model organisms

**Figure 9:**
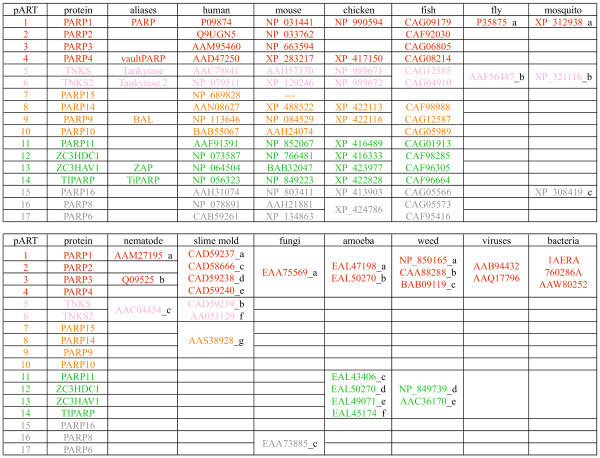
**pARTs in distantly related species. **pART relatives were identified by PSI-BLAST searches as in Figure 4. Matching sequences from other organisms were sorted by group on the basis of sequence similarity and associated domains. Accession numbers are given for pARTs from *Homo sapiens *(human), *Mus musculus *(mouse), *Gallus gallus *(chicken), *Tetraodon nigroviridis *(puffer fish), *Drosophila melanogaster *(fruit fly), *Anopheles gambiae *(malaria mosquito), *Caenorhabditis elegans *(nematode), *Dictyostelium discoideum *(slime mold), *Gibberella zeae *(ear root microfungus), *Entamaoeba histolytica *(amoeba), *Arabidopsis thaliana *(cress plant), Chilo iridescent virus and Bacteriophage Aeh1 (viruses), *Pseudomonas aeruginosa*, *Corynebacterium diphtheriae *and *Vibrio cholerae *(bacteria). Lower case letters in black indicate the pART designations used in Figure 8.

We also used PSI-BLAST to screen the protein database for recognizable pART family members in other organisms using as queries the amino acid sequences of catalytic domains of each of the 17 human pARTs (Figure 9). The order in which PSI-BLAST picked up putative pART sequences from the database in successive iterations was similar for different members of a particular pART subgroup but differed markedly for members of different subgroups (see additional file 8: "Representative tiling paths of PSI-BLAST searches initiated with the catalytic domain amino acid sequences of selected pART family members"). In many instances, PSI-BLAST detected pART sequences from distantly related organisms in earlier iterations than the human pART paralogues from other subgroups.

Figure 9 summarizes the matches of pART-related proteins found in model organisms with completed genome sequences. On the basis of amino acid sequence similarity, conserved intron positions and/or conserved associated domains, pARTs from other vertebrates including fish and chicken, generally can be assigned to a particular human pART orthologue. In contrast, pARTs of lower eucaryotes can be assigned to a subgroup but not to a particular vertebrate pART.

pART homologues were found in many model organisms from the animal, plant, fungi, and protist kingdoms. The recently completed genome of the pufferfish *T. nigroviridis *contains recognizable orthologues for all pARTs except for pART7 [43]. The nearly completed albeit still fragmentary chicken genome contains recognizable orthologues for all pARTs except for pARTs 2, 3, 7, 10, and 17 [44]. Simpler eucaryotes generally contain fewer pARTs (two in the fruit fly *D. melanogaster*, three each in the malaria mosquito *A. gambiae*, the nematode *C. elegans*, and the ascomycete *G. zeae*; six in the amoeba *E. histolytica*, nine in the slime mold *D. discoideum*, and ten in the cress plant *A. thaliana*).

Remarkably, the yeast *S. cerevisae *and the archaea lack detectable pARTs. Only two matches were found in the viral proteome: these derive from two double stranded DNA viruses: the insect virus Chilo iridescent virus and the bacteriophage Aeh1. Although PSI-BLAST initially failed to connect the pART family with Diphtheria toxin and Pseudomonoas exotoxin A, these toxins were readily connected with the eucaryotic pARTs when using as query a chimera, e.g. of Diphtheria toxin and Chilo iridescent virus pART in which the sequences of three of the conserved structure units highlighted in Figures 1 and 6A were interchanged. These searches uncovered a DT/ETA-like putative ADP-ribosyltransferase in *V. cholerae*, but no other proteins in the microbial proteome in GenBank.

Of note, none of the known R-S-E motif bacterial or vertebrate mARTs were ever connected by PSI-BLAST with the DT/ETA/pART group. In several cases, however, we observed intriguing matches just slightly below threshold (in the region surrounding the conserved H in β 1) to members of the family of RNA:NAD 2' phosphotransferases. These enzymes catalyze a reaction during tRNA splicing that is similar to the reaction catalyzed by ARTs, but in which ADP-ribose is transferred to the 2'-phosphate in immature tRNA rather than to an amino acid residue in a protein [25]. The 3D-structure of a prototype member of this gene family, indeed, reveals a structure closely resembling that of PARP-1 and Diptheria toxin (see Fig. 1), providing strong support for the relevance of the matches detected by PSI-BLAST.

For the pART homologues shown in Figure 9 we also analyzed the sequences flanking the pART catalytic domain for associated conserved domains. The results reveal that many pARTs, even from very distantly related organisms, share domain associations found in human and mouse pARTs. Some of these are illustrated in Figure 8. For example, the association of regulatory, BRCT, and DNA binding domains observed in pART1 (PARP-1) is found also in similar proteins encoded by fruit fly, nematode, microfungi and cress plant genomes. Tankyrase-like association with ankyrin repeats is found in pARTs from the fruit fly and nematode. The association of a pART catalytic domain with an A1pp domain, as seen in human pART subgroup 3, is found also in a pART from the slime mold *Dictyostelium discoideum*. The combination with a WWE domain, as seen in human pART subgroup 4, is found also in putative pARTs from cress plant. A domain corresponding to the unknown upstream region of the smallest human pART (pART15) is observed also in a pART from the malaria mosquito *Anopheles gambiae*, and a duplicated truncated pART catalytic domain as in pARTs 16 and 17 is observed also in a pART from the microfungus *Gibberella zeae*. These results indicate that many of the domain combinations observed in human and mouse pARTs represent evolutionary ancient inventions.

Some pARTs of distantly related proteins are associated with domains not found in any of the human pARTs. A striking example is that of *G. zeae *pARTc, which most closely resembles human pARTs 16 and 17, but is associated with a second potential catalytic, ubiquitin ligase domain (Fig. 8). A similar pART is found also in the related microfungus *Aspergillus nidulans *[GenBank: EAA66581]. These microfungal pARTs are the only examples found so far, in addition to vertebrate pART4, where a distinct domain(s) is genetically fused to the C-terminal end of the pART catalytic domain. The large domain(s) associated with the putative pART from bacteriophage Aeh1 does not bear any resemblance to pART-associated domains in vertebrates but shows distant similarity to viral coat proteins. The only organism containing an isolated pART domain reminiscent of the isolated ART domain found in verbetrate mARTs [27] is the Chilo iridescent insect virus. This "naked" viral pART catalytic domain contains the H-Y-E motif of PARP-1 and DT. It will be interesting to determine whether this protein exhibits the predicted pART activity.

A striking example of domain shuffling is observed in one of the three *C. elegans *pARTs: like the human tankyrases (pARTs 5 and 6), Ce.pARTc contains ankyrin repeats, but also harbors the regulatory and WGR domains typical of human group 1 pARTs instead of the SAM domain found in human pARTs 5 and 6 (Fig. 8). A similar variation of domains as in Ce.pARTc is found also in one of the ten pARTs of *D. discoideum *(Dd.pARTb).

**Figure 10:**
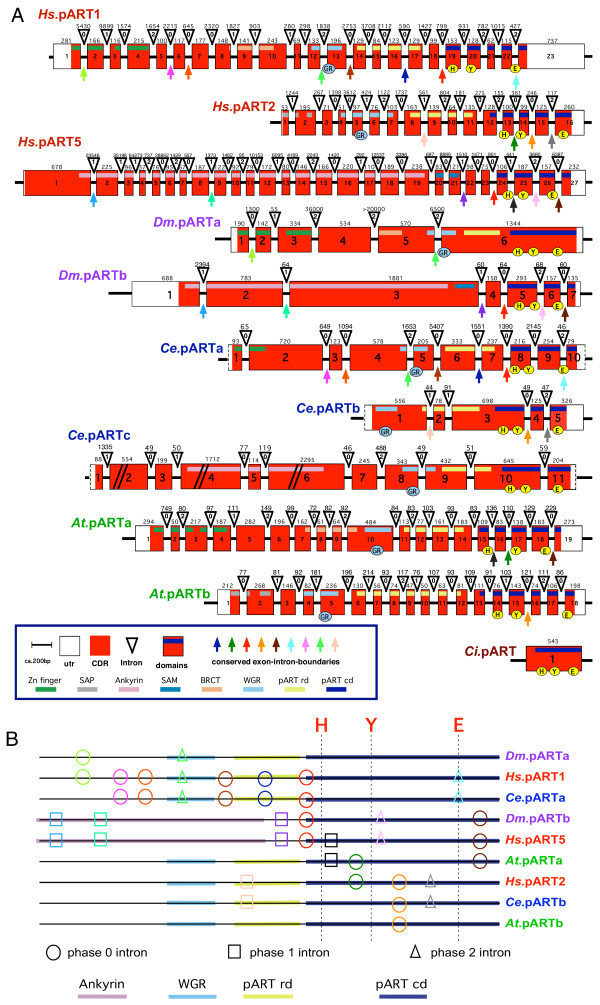
**Schematic diagram of the exon/intron structures of pART family members of distantly related organisms. **A) Exon/intron structures were determined by BLASTn searches of the genome browsers using the pART cDNA sequences. The positions of codons corresponding to the H Y E motif in the NAD-binding crevice of diphtheria toxin are marked by yellow circles. The position of the conserved glycine and arginine pair of residues within the WGR domain is marked in blue. Coding regions for catalytic and other domains are indicated by colored bars. Conserved introns are marked by colored arrows. B) The diagram contains only those introns that are conserved in at least two distantly related species. Color-coding of the introns corresponds to that shown in A). The position of codons encoding/corresponding to the H, Y, E residues in the NAD binding crevice are indicated by vertical lines. The position of each intron with respect to the codon is indicated by circles (phase 0 introns), boxes (phase 1 introns), and triangles (phase 2 introns). Coding regions for catalytic and other selected domains are indicated by colored lines as in A).

Finally, we addressed the question whether the striking differences in exon/intron compositions of the closest PARP-1-homologues in groups 1 and 2 might be reflected in similar differences in pART orthologues of distantly related species. To this end we determined the exon/intron structures of distant pART orthologues by BLASTn searches of the respective genome databases using cDNA sequences as queries; and compared the results with those obtained for human pART genes. The results are illustrated schematically in Figure 10, with conserved intron positions highlighted. As in case of most other genes, the pART genes of 'lower' animals, protists, and plants in general contain fewer and shorter introns than the human homologues. However, some of the introns found in human pART genes are found also in homologues of distantly related organisms. For example, all six introns observed in *D. melanogaster *pARTb are found in corresponding positions also in human pART5 (tankyrase 1); yet human pART5 contains 14 additional introns not found in the fruit fly pART. The other pART of the fruit fly shares two of its five introns with human pART1 (PARP-1). The three pARTs of the nematode *C. elegans *show a different, only partially overlapping set of conserved introns: Ce.pARTa shares seven of its nine introns with human pART1, Ce.pARTb shares three of its four introns with human pART2, whereas Ce.pARTc does not seem to share any of its introns with pART5, despite the similar domain organization on the protein level (see Fig. 8). The pARTs from the model plant *Arabidopsis thaliana *contain a fairly high number of introns, however only very few intron positions correspond to ones found also in human pARTs. For example, At.pARTa which is most closely related to human PARP-1 in terms of amino acid sequence similarity and organization of conserved protein domains, evidently does not share any of its 18 introns with human pART1. Strikingly, however, the introns found in the catalytic domain of this pART exhibit conserved positions with two different human pARTs: two of the four intron positions in the catalytic domain of At.pARTa are found in corresponding positions in human pART5 (tankyrase), another intron is found at a corresponding position in human pART2 (Fig. 10), whereas the fourth intron is not found in any human pART. At.pARTb which is most closely related to human pART2 in terms amino acid sequence similarity and domain organization, shares one of its 17 introns with human pART2. Note further, that in only two cases (Chilo iridescent virus pART and pARTa of the fruit fly), the pART catalytic domain lacks introns, i.e. is encoded by a single exon as in case of the vertebrate mARTs [27].

## Discussion

The results of our study illustrate the great power and utility of the public genome databases and database search programs. Moreover, they provide important novel insights into the molecular structure and evolution of the pART gene family.

Our results differ in some details from those of a recent report by Ame and coworkers [11]. These discrepancies can be explained by errors in the draft sequence of the human genome available at the time of the previous report. For example, the database entry AK023746 given by Ame *et al*. for PARP-5c evidently represents a truncated cDNA for pART6 (alias tankyrase 2 or PARP-5b). This entry contains two point mutations and a 65 bp deletion in the 3' utr vs. the cDNA and genomic sequences of pART6. Blast analyses of the high quality sequence of the human genome and of the EST database with the AK023746 sequence provide no evidence for a distinct copy of this gene in the human genome. We conclude that the PARP-5c gene identified by Ame *et al*. represents an allelic variant or cloning/sequencing error rather than a genuine pART gene family member; i.e. that the total number of human pART genes is 17 rather than 18 suggested in the previous report. Large discrepancies exist also in the number of amino acids assigned in the two reports for pART7/PARP-15 (444 *vs*. 989) and for pART16/PARP-8 (854 *vs*. 501). The earlier database entries for PARP-8 (XM_018395) and PARP-15 (XM_093336) have hence been removed as a result of standard genome annotation processing because these entries evidently contained frameshift mutations and/or fused cDNA sequences that led to erroneous amino acid assignments. Similarly, the small differences in assignments for five other PARPs/pARTs can be accounted for by differences in the draft vs. high quality sequence of the human genome (Ame *et al*./our study): pART2/PARP2 (583/570), pART3/PARP3 (540/533), pART10/PARP10 (1020/1025), and pART14/PARP7 (657/680).

We assigned the 17 human pARTs into five distinct subgroups (Fig. 2). This assignment is supported by several independent lines of evidence: Firstly, members of a particular subgroup show higher amino acid sequence identities to one another than to members of other subgroups (Fig. 6). This is reflected in the tiling paths of PSI-Blast searches, where members of the same subgroup were detected in the first iteration, whereas members of other subgroups generally were detected in later iterations (Fig. 4). Secondly, members of a particular subgroup typically share one or more associated domains not found in members of other subgroups (Fig. 8); pARTs 8, 10 and 15 pose exceptions to this rule. Thirdly, members of a particular subgroup typically share one or more intron positions not found in members of other subgroups (Fig. 3); pARTs 1–4 pose notable exceptions to this rule. Fourthly, when genes of two or more pARTs are physically linked in a cluster on the same chromosome, they belong to the same subgroup – possibly reflecting regional duplications (Fig. 2). Finally, results of all phylogenetic analysis converged in topologies with clearly distinct clades for each of the subgroups (Fig. 7). Members of subgroups 1 and 2 evidently are more closely related to one another than to other subgroups (Figs. 6 and 7). Similarly, members of subgroups 3 and 4 are sister-groups to one another, indicating a close relationship.

Members of the pART family are found fused to a striking variety of associated domains (Fig. 8). It is not farfetched to hypothesize that the associated domains direct the respective pARTs to subcellular structures and/or target proteins. Genetic fusion of group 1 and group 2 pARTs with DNA-binding domains is in line with their established roles in DNA-repair, chromosome remodeling, and mitotic spindle formation [9,11,12]. Moreover, the SAM and ankyrin domains of pARTs 5 and 6 have been shown to mediate interactions with target proteins in telomere-associated protein complexes [45]. Similarly, the C-terminal domain of pART4 evidently plays a role in targeting pART4 to the major vault particles [46]. A flurry of domains implicated in the ubiquitination pathway point to a possible connection between ubiqutitination and ADP-ribosylation. Indeed, it has recently been reported that ADP-ribosylation of TRF1 by tankyrase (pART5) results in the release of the protein from telomers and its subsequent ubiquitination [47]. Strikingly, pARTs from the microfungi *G. zea *and *A. nidulans *provide examples for the genetic fusion of two enzyme domains catalyzing these post-translational protein modifications into a single polypeptide.

So far, only a single example of a 'naked' pART catalytic domain akin to the isolated catalytic domain of the vertebrate ecto-ARTs 1–5 [27] was recovered from the public database. This putative pART from Chilo iridescent virus clusters with the mammalian pARTs of subgroup 1 (Fig. 7), suggesting that this large double stranded DNA virus [48] may have acquired its pART by horizontal gene transfer.

The definition of the pART catalytic domain proposed in this paper is somewhat smaller than that commonly used in the field [11]. We used the position of the common phase 0 intron upstream of the first conserved β sheet to set the N-terminal end of the catalytic domain (e.g. see Figs. 1 and 3B). The pARTs of subgroup 1 are extended N-terminally of this position by an alpha helical domain (Fig. 8) which is often included as part of the PARP-1 catalytic domain. However, since other pART family members lack this region, we propose to omit it from the proper pART catalytic domain. Moreover, this N-terminal delineation of the catalytic domain corresponds well to the N-terminus of the 'naked' pART of Chilo iridescent virus as well as to those of Diphtheria toxin and Pseudomonas exotoxin A after proteolytic processing of the signal sequence or translocation domain (Fig. 1).

With the exception of pART4, the group 1 pARTs are extended upstream of this helical region by another domain named after its conserved motif of tryptophane (W) – glycine (G) – arginine (R) residues. This WGR domain is found also in poly-A-polymerases, its function is unknown. Many group 1 pARTs from distantly related organisms, e.g. plants, insects, nematodes, and microfungi, also contain these two domains. Interestingly, in *Drosophila melanogaster *pARTa these three domains (WGR, helical, catalytic) are encoded by a single, large exon (Fig. 10). Human pARTs 5–17 lack the WGR and helical domains. However, pART5/6 (tankyrase)-like pARTs from *C. elegans *(Ce.pARTc) and *D. discoideum *(Dd.pARTb) contain the WGR and helical domains whereas a SAM domain is found at this position in human pARTs 5 and 6 (Fig. 8).

A puzzling finding is the lack of conservation of the classic H-Y-E motif found in the catalytic cores of PARP-1, PARP-2, Diphtheria toxin and Pseudomonas Exotoxin A (Fig. 1). This motif is conserved only in members of subgroups 1 and 2. All other human pARTs carry notable variations from this motif. In particular, all other pARTs carry a replacement of the glutamic acid residue in β 5, i.e. the residue that was shown to be critical for the catalytic activities of DT, PARP-1 and many other pARTs and mARTs [6,7,20,21]. In six cases, this glutamic acid is replaced by an isoleucine residue, in two cases by leucine, and in one case each by threonine, valine, or tyrosine. Enzyme activity has been reported recently for two of the six pARTs that carry an H-Y-I motif instead of the H-Y-E motif (pARTs 10 and 14) [32,34]. Thus, it is not unlikely that the four other pARTs carrying the H-Y-I motif turn out to be active enzymes (pARTs 11, 12, 16, and 17). Mouse pART8 also carries an H-Y-I motif, whereas its human orthologue, like pART7, carries an H-Y-L variant motif. H-Y-I and H-Y-L variant motifs are also found in pARTs from the slime mold (Dd.pARTg) and amoeba (Eh.pARTf) (Fig. 8). Human pART15 carries an H-Y-Y variant motif, which is conserved in its orthologues from mouse and the malaria mosquito (Fig. 8). It will be interesting to determine whether and how site directed mutagenesis of the H-Y-E motif in pARTs 1–6 to the variant motifs of pARTs 7–17 – and vice versa – affects their enzyme activities. Moreover, it remains to be determined whether the most striking variation of the H-Y-E motif – to Q-Y-T in human and mouse pART9 is compatible with enzyme activity.

The results of our PSI-BLAST and PSIPRED analyses (Figs. 4, 5, 9 and additional files 3, 4, 5, 6, 7, 8) support the conclusions that the pART gene family described here and the mART gene family described in our previous study [27] constitute two distinct ART subfamilies, and further, that the family of tRNA:NAD 2'-phosphotransferases [24,25] constitutes a branch that is more closely related to the pART subfamily than to the mART subfamily. Our results illuminate the power and limits of PSI-BLAST searches: PSI-BLAST readily connected members of the pART subfamily in many different species, while DT, ETA and TpTs were found at or below the threshold. In contrast PSI-BLAST searches never connected pART family members with members of the mART subfamily or vice versa. The results of PSI-BLAST searches, thus, are in accord with insights gained from the known 3D structures of representative ADP-ribosyltransferases (Fig. 1), i.e. that certain conserved structural features clearly distinguish these two subfamilies. Is it possible that some of the pART gene family members described here actually possess mono-ADP-ribosyltransferase rather than poly-ADP-ribosyltransferase activity? Given the structural similarity to DT/ETA this is a possibility. Moreover, it cannot be excluded that some family members may have lost enzyme activity and have acquired a new function. In any case, the respective proteins clearly are more closely related to the pART than to the mART gene family, in line with the nomenclature proposed here. Have all ARTs encoded in the human genome been identified? A number of ADP-ribosylation reactions have been described in mammalian cells that cannot yet be accounted for by the ARTs identified in this study or our previous study, e.g. mono-ADP-ribosylation of actin, rho, glutamate dehydrogenase, and of the alpha and beta subunits of heterotrimeric G proteins [3,4,8]. Given the fact that the pART subfamily described here and the mART subfamily described in our previous study [27] could not be interconnected by PSI-BLAST, it reamins an intriguing possibility that other ART subfamilies in the human genome still await to be identified.

## Conclusion

The family of proteins containing a PARP-like catalytic domain consists of 17 members in the human and 16 in the mouse, rat, and pufferfish. The vertebrate pART family can be divided into five subgroups on the basis of sequence similarity, phylogenetic relationships, conserved intron positions, and patterns of genetically fused protein domains. The four members of group 1 and the two members of group 2 each contain a conserved trias of residues (H-Y-E motif) also observed in Diphtheria toxin and Pseudomonas exotoxin A. The eleven other pART proteins carry variants of this motif (six H-Y-I, two H-Y-L, and one each Q-Y-T, Y-Y-V, H-Y-Y). All human pARTs are multi-domain proteins in which the pART catalytic domain is associated in a Lego-like fashion with other putative protein-protein interaction, DNA binding and ubiquitination domains. In all but one case (pART4) the catalytic domain represents the C-terminal end of the multi-domain protein. Most of the domain associations observed in human pARTs appear to be very ancient inventions since they can be found also in insects, plants, microfungi, and amoeba.

## Methods

### Database searches

Protein databases were searched using PSI-BLAST [35]. Genome databases were searched using BLASTn and tBLASTn [49]. Tissue distributions of pART-ESTs were analyzed using Electronic Northern calculations at the GeneCard website [50].

### Structure and sequence analyses

Amino acid sequence alignments were performed with T-Coffee [36]. Secondary structure predictions were performed with PSIPRED [37]. Threading of amino acid sequences onto known 3D structures in PDB were performed with GenTHREADER [37]. Sequence analyses were performed using DNA-Star software, 3D-images were prepared with PyMol [51] software.

### Phylogenetic analyses

Phylogenetic analyses were applied to the 36 catalytic core amino acid sequences using the dataset in Figure 6. Phylogenetic analyses were performed on the computational cluster of the College of Biology and Agriculture at Brigham Young University by using maximum parsimony and Bayesian Markov chain Monte Carlo approaches . The topologies were reconstructed using equally weighted maximum parsimony (MP) analysis as implemented in PAUP* 4.0b10 [52], maximum likelihood (ML) with simultaneous adjustment of topology, and branch length as implemented in PhyML [53], as well as Bayesian methods coupled with Markov Chain Monte Carlo inference (BMCMC, MrBayes) [54]. The best fit likelihood model for amino acid evolution was determined based on the lowest Akaike Information Criterion (AIC) or Bayesian Information Criterion (BIC) score as implemented in ProtTest 1.2.6 [53,55,56].

The MP analysis was run using 5000 random addition replicates and tree bisection-reconnection branch swapping. Nonparametric bootstrap values were calculated for MP and ML analyses (10.000/100 bootstrap replicates, 100/1 heuristic random addition replicates) to assess confidence in the resulting relationships. ML analysis was run implementing the RtREV+I+G+F model of amino acid evolution (AIC= 4907.73; -lnL= 2800). The *a priori *information obtained by ProtTest 1.2.6 was incorporated into the BMCMC analysis. Bayesian phylogeny estimation was achieved using random starting trees, run for 3 × 10^6 ^generations, with a sample frequency of 1000, and ten chains (nine heated, temperature= 0.2). Analyses were repeated three times to check for likelihood and parameter mixing and congruence. Likelihood scores were plotted against generation time to determine stationery levels. Sample points before reaching stationery were discarded as "burn-in". Repeated analyses were compared for convergence on the same posterior probability distributions [57]. The maximum *a posteriori *tree (MAP) is presented in this paper, showing to percentage converted posterior probabilities (p*P*%).

## Abbreviations used

ART = ADP-Ribosyltransferase, BLAST = basic local alignment search tool, 3MB = 3-methoxybenzamide, NAD = nicotinamide adenine dinucleotide, PDB = protein database.

## Authors' contributions

This study was initiated in the summer of 1997 while FKN was a visiting scientist in FB's lab at DNAX. Initial database searches were performed by FKN and FB, later searches by HO, PAR, and FKN. KD performed the phylogenetic analyses. FKN supervised the study with essential contributions by FH. HO prepared the figures and FKN wrote the paper. The results represent the partial fulfillment of the requirements for the graduate thesis of HO.

## Supplementary Material

Additional File 1 **Representation of pART gene transcripts in the database of expressed sequence tags **The public EST database was screened for ESTs encoding pARTs using tBLASTn and the amino acid sequences of the catalytic domain of known pART family members as queries at the dates indicated on top. Accession numbers of the corresponding Unigene clusters are indicated. Blank fields indicate lack of detectable ESTs encoding the respective pART catalytic domain. Tissue distribution analyses were performed for each cluster by "electronic Northern" analyses. For each family member, the two tissues with the highest numbers of ESTs are indicated. Tissue abbreviations: BMR bone marrow, BRN brain, HRT heart, MSL muscle, PNC pancreas, PST prostate, KDN kidney, LNG lung, LVR liver, LYN lymph node, SPC spinal chord, SPL spleen, TMS thymus, UTR uterus Click here for file

Additional File 2 **Schematic illustration of the local human and mouse chromosomal environments of the pART subgroup 3 gene cluster **The figure schematically illustrates the local chromosomal environment of the syntenic cluster of *pART *genes and neighboring genes on human chromosome 3q (top) and mouse chromosome 16B3 (bottom). The order and orientation of all genes in the depicted cluster is conserved. Known transcripts in GenBank are indicated schematically with their respective accession number. Exons are indicated by boxes. The direction of transcription is marked by arrows. Grey vertical bars correspond to a scale of 10.000 base pairs. The figure was modified from the respective online UCSC human and mouse genome browsers . Click here for file

Additional File 3 **Multiple amino acid sequence alignments, secondary structure predictions, and threading results for pART subgroup 1 **A multiple sequence alignment was generated for the catalytic domains of pARTs 1–4 with T-Coffee. Each residue in the sequence is reported as a single letter code. Secondary structure units in the 3D structures of chicken PARP-1 (1a26) and mouse PARP-2 (1GS0) are indicated on top of the alignment. Positions with identical residues in all sequences are marked by asterisks, similarities are marked with colons and periods below the alignment. Residues corresponding to the H Y E motif in the NAD binding crevice of diphtheria toxin are marked in red. Intron positions are projected onto the multiple alignment and are marked in grey (phase 0), blue (phase 1), and yellow (phase 2). Secondary structure predictions were generated for human pART1 with PSIPRED and are indicated in blue below the alignment (pr1); the confidence of the prediction is indicated in orange (highest confidence = 9). Secondary structure units are abbreviated as follows: H = helix; B = residue in isolated beta bridge; E = extended beta strand; G = 310 helix; I = pi helix; T = hydrogen bonded turn; S = bend. Click here for file

Additional File 4 **Multiple amino acid sequence alignments, secondary structure predictions, and threading results for pART subgroup 2 **A multiple sequence alignment was generated for the catalytic domains of pARTs 5 and 6 with T-Coffee. Residues, identities, intron positions, and secondary structure units are marked as in additional file 3. Indicated secondary structure predictions were generated for human pART5 (pr5) with PSIPRED. Click here for file

Additional File 5 **Multiple amino acid sequence alignments, secondary structure predictions, and threading results for pART subgroup 3 **A multiple sequence alignment was generated for the catalytic domains of pARTs 7–10 with T-Coffee. Residues, identities, intron positions, and secondary structure units are marked as in additional file 3. Indicated secondary structure predictions were generated for human pART7 (pr7) with PSIPRED. Click here for file

Additional File 6 **Multiple amino acid sequence alignments, secondary structure predictions, and threading results for pART subgroup 4 **A multiple sequence alignment was generated for the catalytic domains of pARTs 11–14 with T-Coffee. Residues, identities, intron positions, and secondary structure units are marked as in additional file 3. Indicated secondary structure predictions were generated for human pART11 (pr11) with PSIPRED. Click here for file

Additional File 7 **Multiple amino acid sequence alignments, secondary structure predictions, and threading results for pART subgroup 5 **A multiple sequence alignment was generated for the catalytic domains of pARTs 15–17 with T-Coffee. Residues, identities, intron positions, and secondary structure units are marked as in additional file 3. Indicated secondary structure predictions were generated for human pART15 (pr15) and for human pART16 (pr16) with PSIPRED. Click here for file

Additional File 8 **Representative tiling paths of PSI-BLAST searches initiated with the catalytic domain amino acid sequences of selected pART family members **PSI-BLAST searches were initiated with the query sequences indicated on top at a threshold setting for the expect value of 0.005 as in Figure 4. pART subgroups are color coded as in Figure 2. Matching sequences from the slime mold (*D. discoideum*, blue) and from a model plant (*A. thaliana*, green) are indicated at the iteration in which they first appeared above threshold. The respective pART homologues from these species were arbitrarily numbered (pARTa-j) in the order in which they were detected in the search that was initiated with human pART1 (PARP-1). Protein data base accession numbers are listed in Figure 9. pARTs indicated in black include short possibly truncated coding sequences of pART homologues that could not be assigned to a particular subgroup with certainty. Click here for file
