# Supplementary material for: In silico characterization of the family of PARP-like poly(ADP-ribosyl)transferases (pARTs)
Source: BMC Genomics. 2005 Oct 4;6:139. doi: 10.1186/1471-2164-6-139 (PMC1266365; doi:10.1186/1471-2164-6-139)
Supplement: Additional File 1 — Representation of pART gene transcripts in the database of expressed sequence tags The public EST database was screened for ESTs encoding pARTs using tBLASTn and the amino acid sequences of the catalytic domain of known pART family members as queries at the dates indicated on top. Accession numbers of the corresponding Unigene clusters are indicated. Blank fields indicate lack of detectable ESTs encoding the respective pART catalytic domain. Tissue distribution analyses were performed for each cluster by "electronic Northern" analyses. For each family member, the two tissues with the highest numbers of ESTs are indicated. Tissue abbreviations: BMR bone marrow, BRN brain, HRT heart, MSL muscle, PNC pancreas, PST prostate, KDN kidney, LNG lung, LVR liver, LYN lymph node, SPC spinal chord, SPL spleen, TMS thymus, UTR uterus [file 1471-2164-6-139-S1.pdf]

gene	number of ESTs in cluster						Unigene cluster	tissues
	0ct 99	Jun 01	Jun 03	Feb 02	0ct 03	0ct 04		
A) pARTs								
1	157	339	492	527	756	862	Hs.177766	PNC / LNG
2	54	58	78	84	120	122	Hs.409412	SPL / LNG
3	23	24	40	64	109	109	Hs.271742	LNG / LVR
4	106	132	187	202	282	337	Hs.117825	LNG / TMS
5	81	108	181	188	260	298	Hs.370267	PST / BRN
6			129	134	205	430	Hs.329327	SPC / MSL
7		7	10	12	8	12	Hs.120250	LYN / UTR
8		10	241	252	298	331	Hs.518203	LNG / BMR
9			122	132	226	243	Hs.518200	LNG / PNC
10	47	25	67	152	279	290	Hs.348609	SPL / TMS
11		10	12	16	33	99	Hs.504538	BMR / PST
12	51	67	115	134	183	187	Hs.12646	KDN / PST
13		62	93	96	288	312	Hs.133512	BMR / SPL
14	39	87	140	145	196	214	Hs.12813	LVR / LNG
15					107	116	Hs.30634	TMS / PST
16			61	64	121	166	Hs.369581	TMS / SPL
17			161	175	286	304	Hs.270244	SPL / BRN
B) other								
PARG	28	34	73	88	82	77	Hs.91390	BMR / LVR
HPRT	46	67	148	178	273	299	Hs.412707	SPL / KDN
GAPD	>2000	>3700	>10000	>10000	>15000	>15000	Hs.479728	MSL / HRT
ART1	0	0	0	0	1	1	Hs.382188	MSL
ART2P	0	0	0	0	0	0	--	--
ART3	21	35	66	74	87	95	Hs.24976	MSL / HRT
ART4	9	9	11	11	16	23	Hs.13776	LVR / KDN
ART5	3	4	3	10	13	17	Hs.125680	LVR / MSL
