# Supplementary figures and images for: In silico characterization of the family of PARP-like poly(ADP-ribosyl)transferases (pARTs)
Source: BMC Genomics. 2005 Oct 4;6:139. doi: 10.1186/1471-2164-6-139 (PMC1266365; doi:10.1186/1471-2164-6-139)

## A human Chromosome 3q

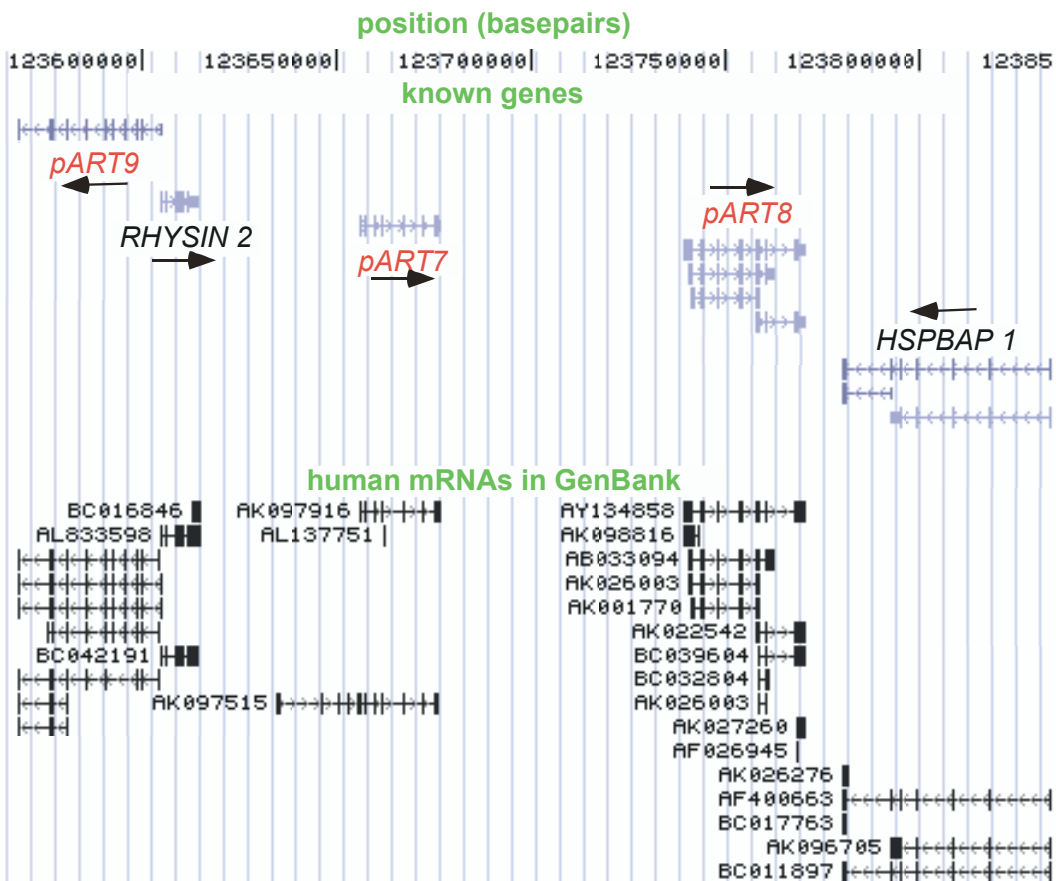

## B murine Chromosome 16B3

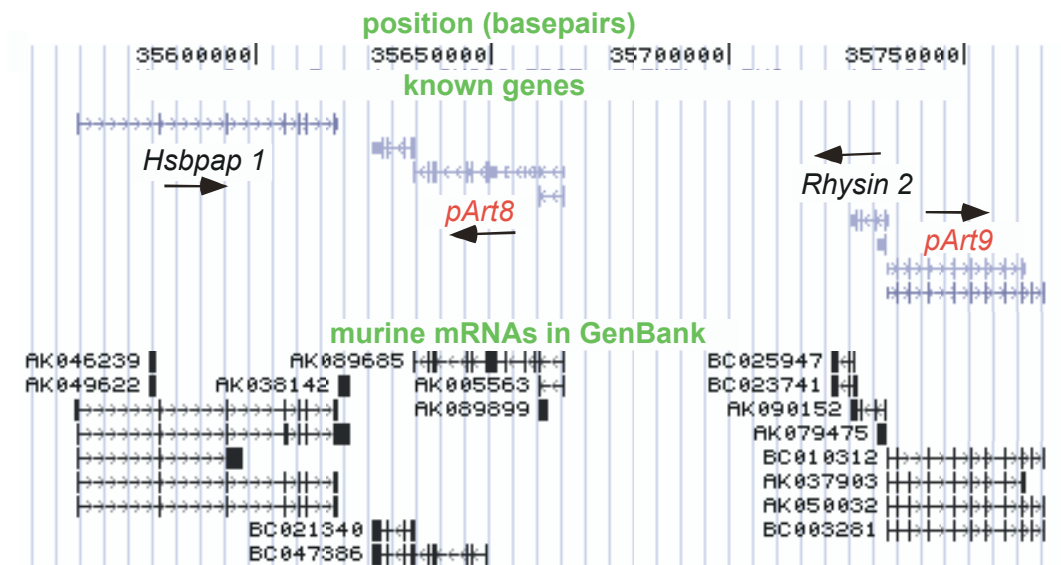

Supplement: Additional File 2 — Schematic illustration of the local human and mouse chromosomal environments of the pART subgroup 3 gene cluster The figure schematically illustrates the local chromosomal environment of the syntenic cluster of pART genes and neighboring genes on human chromosome 3q (top) and mouse chromosome 16B3 (bottom). The order and orientation of all genes in the depicted cluster is conserved. Known transcripts in GenBank are indicated schematically with their respective accession number. Exons are indicated by boxes. The direction of transcription is marked by arrows. Grey vertical bars correspond to a scale of 10.000 base pairs. The figure was modified from the respective online UCSC human and mouse genome browsers . [file 1471-2164-6-139-S2.pdf]
