# Supplementary material for: In silico characterization of the family of PARP-like poly(ADP-ribosyl)transferases (pARTs)
Source: BMC Genomics. 2005 Oct 4;6:139. doi: 10.1186/1471-2164-6-139 (PMC1266365; doi:10.1186/1471-2164-6-139)
Supplement: Additional File 3 — Multiple amino acid sequence alignments, secondary structure predictions, and threading results for pART subgroup 1 A multiple sequence alignment was generated for the catalytic domains of pARTs 1–4 with T-Coffee. Each residue in the sequence is reported as a single letter code. Secondary structure units in the 3D structures of chicken PARP-1 (1a26) and mouse PARP-2 (1GS0) are indicated on top of the alignment. Positions with identical residues in all sequences are marked by asterisks, similarities are marked with colons and periods below the alignment. Residues corresponding to the H Y E motif in the NAD binding crevice of diphtheria toxin are marked in red. Intron positions are projected onto the multiple alignment and are marked in grey (phase 0), blue (phase 1), and yellow (phase 2). Secondary structure predictions were generated for human pART1 with PSIPRED and are indicated in blue below the alignment (pr1); the confidence of the prediction is indicated in orange (highest confidence = 9). Secondary structure units are abbreviated as follows: H = helix; B = residue in isolated beta bridge; E = extended beta strand; G = 310 helix; I = pi helix; T = hydrogen bonded turn; S = bend. [file 1471-2164-6-139-S3.pdf]

```
                                                    β1
1GS0  HHHHHHHHTTTTT  SEEEEE EEEEEEETTHHHHS --TT S EEEEEEE  GGGHHHH
1a26  -----------------------EEEEEETTHHHHHGGGGGSS EEEEEEE  GGGHHHH
1a26  -----------------------IFRIEREGESQRYKPFKQLHNRQLLWHGSRTTNFAGI
hs1   -----------------------IFKIEREGECQRYKPFKQLHNRRLLWHGSRTTNFAGI
mm1   -----------------------IFKIEREGESQRYKPFRQLHNRRLLWHGSRTTNFAGI
hs2   VISQYLQSTHAPTHSDYTMTLLDLFEVEKDGEKEAFR--EDLHNRMLLWHGSRMSNWVGI
mm2   VISQYLQSTHAPTHKDYTMTLLDVFEVEKEGEKEAFR--EDLPNRMLLWHGSRLSNWVGI
hs3   VIQTYLEQT---GSNHRCPTLQHIWKVNQEGEEDRFQAHSKLGNRKLLWHGTNMAVVAAI
mm3   AIQTYLKQT---GNSYRCPDLRHVWKVNREGEGDRFQAHSKLGNRRLLWHGTNVAVVAAI
hs4   --------------SKSPVDVLQIFRVGRVNETTEFL--SKLGNVRPLLHGSPVQNIVGI
mm4   --------------SEQPVDILQIFRVGRVNEATEFL--SKLGNVRLLFHGSPVRNILGI
cons                         ::.: : .*   :    .* *   * **:      .*
pr1   -----------------------CEEEECCCCCHHHHHHCCCCEEEEEEECCCHHHHHHH
conf  -----------------------9156315553367888507984799855985799999

                                  β2     a2                 β3
1GS0  HHH S---    SSS GGG--TTTSSSEEEESSHHHHHGGG   SS--S EEEEEEEEEE
1a26  HHH S---    TTTTGGG--TTT SSEEEESSHHHHHTTS   SS--S EEEEEEEEEE
1a26  LSQGL---RIAPPEAPVTG--YMFGKGIYFADMVSKSANYCHTSQA--DPIGLILLGEVA
hs1   LSQGL---RIAPPEAPVTG--YMFGKGIYFADMVSKSANYCHTSQG--DPIGLILLGEVA
mm1   LSQGL---RIAPPEAPVTG--YMFGKGIYFADMVSKSANYCHTSQG--DPIGLILLGEVA
hs2   LSHGL---RIAHPEAPITG--YMFGKGIYFADMSSKSANYCFASRL--KNTGLLLLSEVA
mm2   LSHGL---RVAPPEAPITG--YMFGKGIYFADMSSKSANYCFASRL--KNTGLLLLSEVA
hs3   LTSGL---RIMPHSG------GRVGKGIYFASENSKSAGYVIGMKCGAHHVGYMFLGEVA
mm3   LTSGL---RIMPHSG------GRVGKGIYFASENSKSAGYVTTMHCGGHQVGYMFLGEVA
hs4   LCRGLLLPKVVEDRGVQRTDVGNLGSGIYFSDSLSTSIKYSHPGET--DGTRLLLICDVA
mm4   LSRGLLLPKVAEDRGVQRTDVGNLGSGIYFSDSLSTSIKYAHAGET--DGSRLLVVCDVA
cons  *  **   ::    .        .*.****:.  *.*  *    .   .    :.: :**
pr1   HHHCC---CCCCCCCCCCC--CEECCEEEECCCHHHHHHCCCCCCC--CCCEEEEEEEEE
conf  87068---67864156346--4210327861300123202136888--870289986553

                             β4
1GS0    SEEEESS  TTGGGGTTT SEEEE  SEE  GGG EEET--T--EEE  S  EE -
1a26    SEEEESS    SS-  TT  EEEE BSEEE TTT EEET--T--EEE    EEE -S
1a26  LGNMYELKNASHITK-LPKGKHSVKGLGKTAPDPTATTTLD--G--VEVPLGNGIST-GI
hs1   LGNMYELKHASHISK-LPKGKHSVKGLGKTTPDPSANISLD--G--VDVPLGTGISS-GV
mm1   LGNMYELKHASHISK-LPKGKHSVKGLGKTTPDPSASITLE--G--VEVPLGTGIPS-GV
hs2   LGQCNELLEANPKAEGLLQGKHSTKGLGKMAPSSAHFVTLN--G--STVPLGPASDT-GI
mm2   LGQCNELLEANPKAQGLLRGKHSTKGMGKMAPSPAHFITLN--G--STVPLGPASDT-GI
hs3   LGREHHINTDNPSLKSPPPGFDSVIARGHTEPDPTQDTELELDGQQVVVPQGQPVPCPEF
mm3   LGKEHHITIDDPSLKSPPSGFDSVIARGQTEPDPAQDIELELDGQPVVVPQGPPVQCPSF
hs4   LGKCMDLHEKDFPLTEAPPGYDSVHGVSQTASVTTD------------------------
mm4   LGKCVNLFKKDFSLTEAPPGYDSVHGVSETTSVPTD------------------------
cons  **.  .:   .        * .*. . ..  . .:
pr1   ECEEEEECCCCHHHC-CCCCCCEEEEECCCCCCCCCCEEEC--C--EEEECCCCCCC-CC
conf  130345307801313-8887650787046678873332226--4--55207888688-75

                  β5          β6
1GS0    SSS   SB EEEES GGGEEE EEEEEEEEE-    ------
1a26  --- S SBSB EEEES GGGEEE EEEEEEEEE     ------
1a26  ---NDTCLLYNEYIVYDVAQVNLKYLLKLKFNYKTS--------
hs1   ---NDTSLLYNEYIVYDIAQVNLKYLLKLKFNFKTSLW------
mm1   ---NDTCLLYNEYIVYDIAQVNLKYLLKLKFNFKTSLW------
hs2   LNPDGYTLNYNEYIVYNPNQVRMRYLLKVQFNF-LQLW------
mm2   LNPEGYTLNYNEFIVYSPNQVRMRYLLKIQFNF-LQLW------
hs3   ---SSSTFSQSEYLIYQESQCRLRYLLEVHL-------------
mm3   ---KSSSFSQSEYLIYKESQCRLRYLLEIHL-------------
hs4   -------FEDDEFVVYKTNQVKMKYIIKFSMPGDQIKDFHPSDH
mm4   -------FQDDEFVVYKTNQVKMKYIVKFCTPGDQIKEFHPHEN
cons         :  .*:::*.  * .::*:::.
pr1   ---CCCCCCCCEEEECCCEEEEEEEEEEEEEECCCCCC------
conf  ---76540455127867864437999999871100279------
```
