# Supplementary material for: In silico characterization of the family of PARP-like poly(ADP-ribosyl)transferases (pARTs)
Source: BMC Genomics. 2005 Oct 4;6:139. doi: 10.1186/1471-2164-6-139 (PMC1266365; doi:10.1186/1471-2164-6-139)
Supplement: Additional File 4 — Multiple amino acid sequence alignments, secondary structure predictions, and threading results for pART subgroup 2 A multiple sequence alignment was generated for the catalytic domains of pARTs 5 and 6 with T-Coffee. Residues, identities, intron positions, and secondary structure units are marked as in additional file 3. Indicated secondary structure predictions were generated for human pART5 (pr5) with PSIPRED. [file 1471-2164-6-139-S4.pdf]

```
                                            ß1
1a26  EEEEEETTHHHHHGGGGGS--------S EEEEEEE  GGGHHHHHHH S    TTTTGG
1a26  IFRIEREGESQRYKPFKQL--------HNRQLLWHGSRTTNFAGILSQGLRIAPPEAPVT
hs5   IQKVVNKKLRERFCHRQKEVSEENHNHHNERMLFHGSP--FINAIIHKGFD---ERHAYI
mm5   IQKVVNKKLRERFCHRQKEVSEENHNHHNERMLFHGSP--FINAIIHKGFD---ERHAYI
hs5   IQKVCNKKLWERYTHRRKEVSEENHNHANERMLFHGSP--FVNAIIHKGFD---ERHAYI
mm5   IQKVCNKKLWERYTHRRKEVSEENHNHANERMLFHGSP--FVNAIIHKGFD---ERHAYI
cons  **** **** **: **:********** *********--*:*********---******
pr5   CCEEECHHHHHHHHHHHHHHHHHHHCCCCCEEEEEECCC--HHHHHHHCCCC---CCCCCC
conf  903206679999999999899987379875377874470--03788844888---466778

                ß2       α2                            ß3
1a26  GTTT SSEEEESSHHHHHTTS---------   SSS---- EEEEEEEEEE  SEEEESS
1a26  GYMFGKGIYFADMVSKSANYC---------HTSQAD----PIGLILLGEVALGNMYELKN
hs5   GGMFGAGIYFAENSSKSNQYVYGIGGGTGCPTHKDRSCYICHRQMLFCRVTLGKSFLQFS
mm5   GGMFGAGIYFAENSSKSNQYVYGIGGGTGCPTHKDRSCYICHRQMLFCRVTLGKSFLQFS
hs5   GGMFGAGIYFAENSSKSNQYVYGIGGGTGCPVHKDRSCYICHRQLLFCRVTLGKSFLQFS
mm5   GGMFGAGIYFAENSSKSNQYVYGIGGGTGCPIHKDRSCYICHRQLLFCRVTLGKSFLQFS
cons  ****************************** ************:***************
pr5   CCCCCCCEEEECCCCCCCCCCCCCCCCCCCCCCCCCCCCCCCCEEEEEEECCCCEEECCC
conf  88123855541244220000025665544554334444333168998860176121157

                   ß4                                      ß5
1a26      SS   TT   EEEE BSEEE TTT EEETTEEE    EEE S   S SBSB EEEES G
1a26  ASHITKLPKGKHSVKGLGKTAPDPTATTTLDGVEVPLGNGISTGINDTCLLYNEYIVYDV
hs5   TMKMAHAPPGHHSVIGR------------------PSVNG---------LAYAEYVIYRG
mm5   TMKMAHAPPGHHSVIGR------------------PSVNG---------LAYAEYVIYRG
hs5   AMKMAHSPPGHHSVTGR------------------PSVNG---------LALAEYVIYRG
mm5   AMKMAHSPPGHHSVTGR------------------PSVNG---------LALAEYVIYRG
cons  :*****:******* **------------------*****---------** ********
pr5   CCCCCCCCCCCCCCCEEC------------------CCCCC---------CCCCEEEEECC
conf  74478888788752546------------------75788---------89870588768

           ß6
1a26  GGEEE EEEEEEEEE   ------------
1a26  AQVNLKYLLKLKFNYKTS------------
hs5   EQAYPEYLITYQIMKPEAPSQTATAAEQKT
mm5   EQAYPEYLITYQIMKPEAPSQTATAAEQKT
hs5   EQAYPEYLITYQIMRPEGMVDG--------
mm5   EQAYPEYLITYQIVRPEGMVDG--------
cons  *************::**.  :
pr5   CEEEEEEEEEEEEEECCCCCCCCCCCCCCC
conf  652068998989822458876644110059
```
