# Supplementary material for: In silico characterization of the family of PARP-like poly(ADP-ribosyl)transferases (pARTs)
Source: BMC Genomics. 2005 Oct 4;6:139. doi: 10.1186/1471-2164-6-139 (PMC1266365; doi:10.1186/1471-2164-6-139)
Supplement: Additional File 5 — Multiple amino acid sequence alignments, secondary structure predictions, and threading results for pART subgroup 3 A multiple sequence alignment was generated for the catalytic domains of pARTs 7–10 with T-Coffee. Residues, identities, intron positions, and secondary structure units are marked as in additional file 3. Indicated secondary structure predictions were generated for human pART7 (pr7) with PSIPRED. [file 1471-2164-6-139-S5.pdf]

```
                                          ß1
1a26  EEEEEETTHHHHHGGGGG--------SS EEEEEEE  GGGHHHHHHH S    TTTT
1a26  IFRIEREGESQRYKPFKQ--------LHNRQLLWHGSRTTNFAGILSQGLRIAPPEAP
hs7   IERIQNAFLWQSYQVKKRQMDIKNDHKNNERLLFHGTDADSVPYVNQHGFNR--SCAG
hs8   IERIQNPDLWNSYQAKKKTMDAKNGQTMNEKQLFHGTDAGSVPHVNRNGFNR--SYAG
mm8   IERIQNPALWRRYQAYKKGMDEKNGNVRNEKHLFHGTEASSLPQLNSNGFNR--SYAG
hs9   VEKIDNEVLMAAFQRKKKMMEEKLHRQPVSHRLFQQVPYQFCNVVCRVGFQR--MYST
mm9   VEQIDNKVLLAAFQEKKKMMEERTPKGSGSQRLFQQVPHQFCNTVCRVGFHR--MYST
hs10  VERVSHPLLQQQYELYRERLLQRCERRPVEQVLYHGTTAPAVPDICAHGFNR--SFCG
mm10  VERVSHPLLQQQYQLHRERLMQSCQQRPVEQVLYHGTSESAVLDICAHGFNR--SFCG
cons  :*::.:  *   ::  :. :     .   .: *:: .       :   **:*--  .
pr7   CCEECCHHHHHHHHHHHHHHHHHHHCCCCCEEEEEECCCHHHHHHHHHHCCCC--CCCC
conf  9120287899999999999998728984306776058978999999826887--7578

                ß2     α2                 ß3
1a26  GGGTTT SSEEEEESSHHHHHTTS   - SSS EEEEEEEEEE  SEEEESS ------
1a26  VTGYMFGKGIYFADMVSKSANYCHT-SQADPIGLILLGEVALGNMYELKNA------S
hs7   KNAVSYGKGTYFAVDASYSAKDTYSKPDSNGRKHMYVVVRVLTGVFTKGRAGLVTPPPK
hs8   KNAVAYGKGTYFAVNANYSANDTYSRPDANGRKHVYYVVRVLTGIYTHGNHSLIVPPSK
mm8   KNATAYGKGTYFAVKASYSACDTYSRPDTNGRKYMYYVVRVLTGNYTNGNASLIVPPSR
hs9   PCDPKYGAGIYFTKNLKNLAEKAKKISAADKLIYVFEAEVLTGFFCQGHPLNIVPPPL
mm9   SYNPVYGAGIYFTKSLKNLADKVKKTSSTDKLIYVFEAEVLTGSFCQGNSSNIIPPPL
hs10  RNATVYGKGVYFAKRASLSVQDRYSPPNADGHKAVFVARVLTGDYGQGRRGLRAPPLR
mm10  RNGTLYGQGVYFAKRASLSVLDRYSPPNAEGYKAVFVAQVLTGDYGQGSRGLKAPPLR
cons       ** * **:   .  .  . . . ::    :: ..**** : :*      **
pr7   CCCCCCCCEEEECCCCCCHHHCCCCCCCCCCEEEEEEEEECCCCCCCCCCCCCCCCC
conf  6777125404531432101101035647887479998753103424787130116767

                ß4                                        ß5
1a26    SS   TT   EEEE BSEEE TTT EEETTEEE    EEE S   S SBSB EEEES G
1a26  HITKLPKGKHSVKGLGKTAPDPTATTTLDGVEVPLGNGISTGINDTCLLYNEYIVYDV
hs7   NPHNPTDLFDSVTN-------------------------------NTRSPKLFVVFFD
hs8   NPQNPTDLYDTVTD-------------------------------NVHHPSLFVAFYD
mm8   DPQNAADLYDTVTD-------------------------------NDKNPSIFVVFYD
hs9   SP-GAIDGHDSVVD-------------------------------NVSSPETFVIFSG
mm9   SP-GALDVNDSVVD-------------------------------NVSSPETIVVFNG
hs10  GPGHVLLRYDSAVD-------------------------------CICQPSIFVIFHD
mm10  VSG-QVLRYDSAVD-------------------------------CLQQPRIFVIFHD
cons   .       *:..:-------------------------------    *  :* * .
pr7   CCCCCCCCCCCCCC-------------------------------CCCCCCEEEEECC
conf  77888876666577-------------------------------8888833788769

          ß6
1a26  GGEEE EEEEEEEEE   ---------------------------------
1a26  AQVNLKYLLKLKFNYKTS---------------------------------
hs7   NQAYPEYLITFTA--------------------------------------
hs8   YQAYPEYLITFRK--------------------------------------
mm8   NQTYPEYLITFRQ--------------------------------------
hs9   MQAIPQYLWTCTQEY-------VQSQDYSSGPMRPFAQHPWRGFASGSPVD
mm9   MQAMPLYLWTCTQDR-TFSQHPMWSQGYSSGPGMVSSLQSWEWVLNGSSV-
hs10  TQALPTHLITCEHVPRASPDDPSGLPGRSPDT-------------------
mm10  TQALPTHLITCKNILPGTP--------------------------------
cons   *: * :* *
pr7   CEECEEEEEEEEC--------------------------------------
conf  6733289777749--------------------------------------
```
