# Supplementary material for: In silico characterization of the family of PARP-like poly(ADP-ribosyl)transferases (pARTs)
Source: BMC Genomics. 2005 Oct 4;6:139. doi: 10.1186/1471-2164-6-139 (PMC1266365; doi:10.1186/1471-2164-6-139)
Supplement: Additional File 6 — Multiple amino acid sequence alignments, secondary structure predictions, and threading results for pART subgroup 4 A multiple sequence alignment was generated for the catalytic domains of pARTs 11–14 with T-Coffee. Residues, identities, intron positions, and secondary structure units are marked as in additional file 3. Indicated secondary structure predictions were generated for human pART11 (pr11) with PSIPRED. [file 1471-2164-6-139-S6.pdf]

```
1a26  EEE  TTSHHHHHHHHHHHHT  STTTTEEEEE EEEEEEETTHHHHHGGGGGSS------
1a26  IKVVDKDSEEAKIIKQYVKNTHAATHNAYDLKVVEIFRIEREGESQRYKPFKQLH------
hs11  LIPLHNQTHEYNEVANLFGKTMDRNR------IKRIQRIQNLDLWEFFCRKKAQLKKKRGV
mm11  LVSLQNQTHEYNEVASLFGKTMDRNR------IKRIQRIQNLDLWEFFCRKKAQLKKKRGV
hs12  KITLSSSSEEYQKVWNLFNRTLPFYF------VQKIERVQNLALWEVYQWQKGQMQKQNGG
mm12  KITLSSSSEEYQKVWNIFNRTLPFYF------VQKIERIQNMGLWEVYQWQKCQMQKQNGG
hs13  LSEIHHLHPEYVRVSEHFKASMKNFK------IEKIKKIENSELLDKFTWKKSQMKEE---
mm13  FIELNNQDEEYAKISEQFKASMKQFK------IVTIKRIWNQKLWDTFERKKQKMKNK---
hs14  QVPVSAEDKSYRIIYNLFHKTVPEFKYR----ILQILRVQNQFLWEKYKRKKEYMNRKMFG
mm14  QVPVSAEDKSYRIIYNLFHKTVPEFKYR----ILQILRVQNQFLWEKYKRKKEYMNRKMSG
cons     :     .*  : . *  ::      ----:  * ::  *  * : :  :*  ::.:
pr11  CCCCCCCCHHHHHHHHHHHHCCCCCE------EEEEEEECCHHHHHHHHHHHHHHHHHHCC
conf  97267788478999999861179833------68988704877889999999999987168

                ß1                                    ß2     α2
1a26  ----- EEEEEEE  GGGHHHHHHH S    TTTTGGGTTT SSEEEEESSHHHHHTTS
1a26  -----NRQLLWHGSRTTNFAGILSQGLRIAPPEAPVTGYMFGKGIYFADMVSKSANYCHTS
hs11  PQ--INEQMLFHGTSSEFVEAICIHNFDWR--INGIHGAVFGKGTYFARDAAYSSRFCKDD
mm11  PQ--INEQMLFHGTSSEFVEAICIHNFDWR--INGVHGAVFGKGTYFARDAAYSSRFCKDD
hs12  KA--VDERQLFHGTSAIFVDAICQQNFDWR--VCGVHGTSYGKGSYFARDAAYSHHYSKSD
mm12  KE--VDERQLFHGTSANFVDAICQQNFDWR--VCGLHGTSYGKGSYFARDAAYSHHYSKSD
hs13  ------GKLLFYATSRAYVESICSNNFDSF--LHETHENKYGKGIYFAKDAIYSHKNCPYD
mm13  -----TEMFLFHAVGRIHMDYICKNNFEWI--LHGNREIRYGKGNYFTKEAMYSHKSCSYD
hs14  RDRIINERHLFHGTSQDVVDGICKHNFDPR--VCGKHATMFGQGSYFAKKASYSHNFSKKS
mm14  RDRIINERHLFHGTSQDVVDGICKHNFDPR--VCGKHATMFGQGSYFAKKASYSHNFSKKS
cons           **:...   :: ** :**:  --:   :   :*:* **::.* **  .  .   .
pr11  CC--CCCEEEECCCHHHHHHHHHHHCCCCC--CCCCCCCCCCCCEEECCCHHHHHHCCCCC
conf  97--21153210688789999999817898--67877872256021100134554046666

                               ß3
1a26  SSS ------------------EEEEEEEEEE  SEEEESS    SS -------  TT  -
1a26  QADP------------------IGLILLGEVALGNMYELKNASHITKL-------PKGKH-
hs11  IKHGNTFQIHGVSLQQRHLFRTYKSMFLARVLIGDYINGDSKYMRPPSKDGSY--VNLYD-
mm11  IKHGNTFQIHGVSLQQRHLFRTYKSMFLARVLIGDYINGDSKYMRPPSKDGSY--VNLYD-
hs12  TQ--------------------THTMFLARVLVGEFVRGNASFVRPPAKEGWS--NAFYD-
mm12  TH--------------------SHMMFLARVLVGDFVRGSTSFVRPPAKEGQS--NAFYD-
hs13  AK--------------------NVVMFVAQVLVGKFTEGNITYTSPPP---------QFD-
mm13  SR--------------------GTVMFVARVLVGSVIEGNMTLSSPPA---------LYD-
hs14  SKG-------------------VHFMFLAKVLTGRYTMGSHGMRRPPPVNPGSVTSDLYD-
mm14  SKG-------------------VHFMFLAKVLTGRYTMGSHGMRRPPPVNPGSVTSDLYD-
cons   :                       **:*:** *    *.     **.          :*-
pr11  CCCCCCCCCCCCCCCCCCCCCCCCEEEEEEECCCCCCCCCCCCEECCCCCCCC--CCCCC-
conf  5666544322222221210136512788521035632588552666898787 5--34457-

        ß4                                     ß5          ß6
1a26  EEEE BSEEE TTT EEETTEEE    EEE S  S SBSB EEEES GGGEEE EEEEEEE
1a26  SVKGLGKTAPDPTATTTLDGVEVPLGNGISTGINDTCLLYNEYIVYDVAQVNLKYLLKLKF
hs11  SCVD-------------------------------DTWNPKIFVVFDANQIYPEYLIDFH-
mm11  SCVD-------------------------------DTWNPKIFVVFDANQIYPEYLIDFH-
hs12  SCVN-------------------------------SVSDPSIFVIFEKHQVYPEYVIQYTT
mm12  SCVN-------------------------------SMSDPTIFVVFEKHQVYPEYLIQYST
hs13  SCVD-------------------------------TRSNPSVFVIFQKDQVYPQYVIEYTE
mm13  SCVD-------------------------------TRLNPSVFVIFRKEQIYPEYVIEYME
hs14  SCVD-------------------------------NFFEPQIFVIFNDDQSYPYFVIQYEE
mm14  SCVD-------------------------------NFFEPQIFVIFNDDQSYPYFVIQYEE
cons  ***:-------------------------------   :* :**:*  .* ** ::*::
pr11  CCCC-------------------------------CCCCCEEEEEECCCEEEEEEEEEEC-
conf  7567-------------------------------8658705898848574137877758-

1a26  EE   -----------------
1a26  NYKTS-----------------
hs11  ----------------------
mm11  ----------------------
hs12  SSKPSVTPSILLALGSLFSSRQ
mm12  SSKPPASPSIFVALGNLFTSRQ
hs13  DKACV------------IS---
mm13  LEKEK---------GCIIS---
hs14  VSNTVSI---------------
mm14  VSNTVSI---------------
cons
pr11  ----------------------
conf  ----------------------
```
