# Supplementary material for: In silico characterization of the family of PARP-like poly(ADP-ribosyl)transferases (pARTs)
Source: BMC Genomics. 2005 Oct 4;6:139. doi: 10.1186/1471-2164-6-139 (PMC1266365; doi:10.1186/1471-2164-6-139)
Supplement: Additional File 7 — Multiple amino acid sequence alignments, secondary structure predictions, and threading results for pART subgroup 5 A multiple sequence alignment was generated for the catalytic domains of pARTs 15–17 with T-Coffee. Residues, identities, intron positions, and secondary structure units are marked as in additional file 3. Indicated secondary structure predictions were generated for human pART15 (pr15) and for human pART16 (pr16) with PSIPRED. [file 1471-2164-6-139-S7.pdf]

```
                                                         β1
1a26  HHHHHHHHHHT  STTTTEEEEE EEEEEEE-TTHH-HHHGGGGGSS EEEEEEE  GGGH
1a26  AKIIKQYVKNTHAATHNAYDLKVVEIFRIER-EGES-QRYKPFKQLHNRQLLWHGSRTTNF
hs15  FEKIQKLTGAPHTPV-PAPDF----LFEIEYFDPAN-AKFYETKGERDLIYAFHGSRLENF
mm15  FEKIQQLTGAPHTPV-PTPDF----LFEIEYFDPAN-SRFYETKGERDLIYAFHGSRLENF
hs16  QLKFM---HTPHQ-------------FLLLSSPPAKESNFRAAKKLFGSTFAFHGSHIENW
mm16  QLKFM---HTPHQ-------------FLLLSSPPAKESNFRAAKKLFGSTFAFHGSHIENW
hs17  -LKFM---HTSHQ-------------FLLLSSPPAKEARFRTAKKLYGSTFAFHGSHIENW
mm17  -LKFM---HTSHQ-------------FLLLSSPPAKEARFRTAKKLYGSTFAFHGSHIENW
cons    *:     :.*   -     ---- * :    **: :.*  :*   .  :*****::**:
pr15  CCHHHHHHCCCCCCC-CCCCC----EEEEEEECCCC-HHHHHHHCCCCCEEEECCCCCHHH
conf  911432006798877-88410----47787605323-578975115784677607621457
pr16  CCCCC---CCCEE-------------EEEECCCHHHHHHHHHHHCCCCCEEEEECCCHHHH
conf  98765---77314-------------77840782111211011057884588626625667

                                   β2    α2
1a26  HHHHHH S    T-TTTGGGTTT SSEEEESSHHHHHTTS   SSS --------------
1a26  AGILSQGLRIAPP-EAPVTGYMFGKGIYFADMVSKSANYCHTSQADP--------------
hs15  HSIIHNGLHCH---LNKT--SLFGEGTYLTSDLSLALIYSPHGHGWQ--------------
mm15  HSIIHNGLHCH---LNKT--SLFGEGTYLTSDLSLALIYSPHGHGWQ--------------
hs16  HSILRNGLVVASNTRLQLHGAMYGSGIYLSPMSSISFGYSGMNKKQKVSA-KDEPASSSKS
mm16  HSILRNGLVVASNTRLQLHGAMYGSGIYLSPMSSISFGYSGMNKKQKVSS-KDEPASSSKS
hs17  HSILRNGLVNASYTKLQLHGAAYGKGIYLSPISSISFGYSGMGKGQHRMPSKDELVQRYNR
mm17  HSILRNGLVNASYTKLQLHGAAYGKGIYLSPISSISFGYSGMGKGQHRMPSKDELVQRYNR
cons  ***::***        :   : :*.* **:.  *::: **  .:  :
pr15  HHHHHHHHEEE---ECCC--EEEEEEEEECHHHHHHHHCCCCCCCCC--------------
conf  99875201024---2051--788634773322301310148998754--------------
pr16  HHHHHCCCCCCCCCCCCCCCCCCCEEEECCCHHHHHHHHCCCCCCCCCCH-HCCCCCCCCC
conf  77773173417788533456510050575350333321000367871110-0000000034

                          β3                         β4
1a26  ----------------EEEEEEEEEE  SEEEESS   SS  TT   EEEE BSEEE TTT
1a26  ----------------IGLILLGEVALGNMYELKNASHITKLPKGKHSVKGLGKTAPDPTA
hs15  HSLLGPILSCVAVCEVIDHPDVKCQTKKKDSKE------------IDRRRARIKH------
mm15  HSLLGPILSCVAVCEVIDHPDVKCQIKKKDSKE------------IDRSRARIKH------
hs16  SNT-SQSQKKGQQSQFLQSRNLKCIALCEVITS------------SDLHKHGE--------
mm16  SNA-SQSQKKGQQSQFLQSRNLKCIALCEVITS------------PDLHKHGE--------
hs17  MNTIPQTRSIQ--SRFLQSRNLNCIALCEVITS------------KDLQKHGN--------
mm17  MNTIPQTRSIQ--SRFLQSRNLNCIALCEVITS------------KDLQKHGN--------
cons   .      .    ...::  :::*    :  ..             *  :
pr15  HHHHHHHHHHHHHHHHCCCCCCCEEECCCCCCC------------CCCCEEEEEE------
conf  013322243311001207876301100102400------------1442113440------
pr16  CCC-CCCCCCCCCCCCCCCCCEEEEEEEECCCC------------CCCEECCC--------
conf  222-44346773201014776103443320178------------66254351--------

                                  β5         β6
1a26   EEETTEEE    EEE S  S SBSB EEEES GGGEEE EEEEEEEEE   ---------
1a26  TTTLDGVEVPLGNGISTGINDTCLLYNEYIVYDVAQVNLKYLLKLKFNYKTS---------
hs15  ------------------SEGGDIPPKYFVVTNNQLLRVKYLLVYSQKPPKRAPSQLSWFS
mm15  ------------------SEGGEIPPKYFVVTNNQLLRVKYLLVYSQKQPKRASSQLSWLS
hs16  ---------------------------IWVVPNTDHVCTRFFFVYEDGQVGDAN-------
mm16  ---------------------------IWVVPNTDHVCTRFFFVYEDGQVGDAN-------
hs17  ---------------------------IWVCPVSDHVCTRFFFVYEDGQVGDAN-------
mm17  ---------------------------IWVCPVSDHVCTRFFFVYEDGQVGDAN-------
cons                              :* . .: : .::::**.:     *
pr15  ------------------CCCCCCCCCEEEECCCEEEEEEEEEEECCCCCCCCCCCCCEEE
conf  ------------------6899888404887078378888888763677757778863012
pr16  ---------------------------EEEECCCCCCCCCEEEEEECCCCCCCC-------
conf  ---------------------------689616764240268986078113452-------

1a26  -----------------------------------
1a26  -----------------------------------
hs15  SHWFTVMISLYLLLLLIVSVINSSAFQHFWNRAKR
mm15  SHWFVIMMSLYLLLLLIVSVTNSSAFHHFWNRVKR
hs16  -----INTQEGGIHKEILRVIGDQTATG-------
mm16  -----INTQEGGIHKEILRVIGNQTATG-------
hs17  -----INTQDPKIQKEIMRVIGTQVYTN-------
mm17  -----INTQDPKIQKEIMRVIGTQVYTN-------
cons       :  .   :   *: * . ..
pr15  EECCHHHHHHHHHHHHHHHHHCCCHHHHHHHHHCC
conf  10000235667776665421143578989987439
pr16  -----CCCCCCHHHHHHHHHHHHCCCCC-------
conf  -----43457336788888750000369-------
```
