# Supplementary material for: In silico characterization of the family of PARP-like poly(ADP-ribosyl)transferases (pARTs)
Source: BMC Genomics. 2005 Oct 4;6:139. doi: 10.1186/1471-2164-6-139 (PMC1266365; doi:10.1186/1471-2164-6-139)
Supplement: Additional File 8 — Representative tiling paths of PSI-BLAST searches initiated with the catalytic domain amino acid sequences of selected pART family members PSI-BLAST searches were initiated with the query sequences indicated on top at a threshold setting for the expect value of 0.005 as in Figure 4. pART subgroups are color coded as in Figure 2. Matching sequences from the slime mold (D. discoideum, blue) and from a model plant (A. thaliana, green) are indicated at the iteration in which they first appeared above threshold. The respective pART homologues from these species were arbitrarily numbered (pARTa-j) in the order in which they were detected in the search that was initiated with human pART1 (PARP-1). Protein data base accession numbers are listed in Figure 9. pARTs indicated in black include short possibly truncated coding sequences of pART homologues that could not be assigned to a particular subgroup with certainty. [file 1471-2164-6-139-S8.pdf]

input	Hs.pART1	Hs.pART9	Hs.pART12	Hs.pART15	Ci.pART
iteration 1 = traditional Blastp searches	At.pARTa		Dd.pARTg		At.pARTb
	At.pARTb		Dd.pARTf		Dd.pARTb
	Dd.pARTa				Dd.pARTa
	Dd.pARTb				
	Dd.pARTc				
	Dd.pARTd				
	At.pARTc				
	Dd.pARTe				
iteration 2	Dd.pARTf	Dd.pARTg	Dd.pARTc	Dd.pARTd	At.pARTa
	Dd.pARTg	Dd.pARTf	At.pARTb		Dd.pARTc
	At.pARTd	Dd.pARTc	At.pARTa		Dd.pARTd
		At.pARTh	Dd.pARTh		At.pARTc
		At.pARTa	Dd.pARTa		Dd.pARTe
		Dd.pARTh	At.pARTg		Dd.pARTf
		At.pARTb	Dd.pARTb		
		Dd.pARTb	At.pARTd		
		At.pARTg	At.pARTf		
		At.pARTf	At.pARTh		
			At.pARTe		
iteration 3	At.pARTe	Dd.pARTa	Dd.pARTd	At.pARTb	Dd.pARTg
	At.pARTf	At.pARTd	Dd.pARTe	At.pARTa	At.pARTd
		At.pARTe	At.pARTi	Dd.pARTa	At.pARTe
		Dd.pARTd	At.pARTc	Dd.pARTb	
		Dd.pARTe	Dd.pARTi	Dd.pARTc	
		At.pARTi		Dd.pARTe	
				Dd.pARTf	
iteration 4	At.pARTg	At.pARTc	At.pARTj	At.pARTc	At.pARTf
	At.pARTh	At.pARTj		Dd.pARTg	At.pARTg
				At.pARTd	At.pARTh
				At.pARTe	
iteration 5	At.pARTi			At.pARTf	At.pARTi
				At.pARTh	
iteration 6	converged			At.pARTg	converged
iteration 7		converged	converged	At.pARTi	
iteration 8				converged	
